# Histologic and combined histologic–endoscopic outcomes with mirikizumab in Crohn’s disease: VIVID-1 trial results

**DOI:** 10.1093/ecco-jcc/jjag077

**Published:** 2026-06-17

**Authors:** Vipul Jairath, Fernando Magro, Marijana Protic, Gert De Hertogh, Noam Harpaz, Tadakazu Hisamatsu, Geert D’Haens, Rish Pai, Guanglei Yu, Nathan Morris, Wen Tao Luo, Emily Hon, Rodrigo Escobar, Luc Biedermann, Walter Reinisch, for the VIVID Study Group, Emiliano Tron, Fernanda Dorado Dorado, Lena Thin, Rupert Leong, Hans Seltenreich, Britt Christensen, Anthony Croft, Simon Ghaly, Robert Koch, Walter Reinisch, Sonja Heeren, Peter Bossuyt, Marc Ferrante, Fabio Luiz Maximiano, Ligia Yukie Sassaki, Carolina Baia, Debora Poli, Alexandre de Sousa Carlos, Carlos Fernando de Magalhaes Francesconi, Alexander De Sa Rolim, Julio Razera, Joaquim Simoes Neto, Rodrigo Rocco, Jonathas Stifft, Jonathan Soldera, Genoile Silva, Cintia Mendes Clemente, Sandra Di Felice Boratto, Luciana Teixeira de Campos, Marcelo Rodrigues Borba, Gregory Rosenfeld, Terry Ponich, Allen Lim, Chadwick Williams, Bruce Musgrave, Stephane Gauthier, Wenjia Liu, Chengdang Wang, Hong Guo, Chunxiao Chen, Yan Chen Chen, Qian Cao, Jie Zhong, Min Xu, Yufang Wang, Changqing Zheng, Xiang Gao, Youxiang Chen, Yinglei Miao, Lin Wang, Deliang Liu, Bangmao Wang, Zhengji Song, Xiaowei Liu, Baili Chen, Yi Jiang, Qiang Zhan, Kaiguang Zhang, Hong Shen, Zhaotao Li, Xiaoyan Wang, Mei Wang Wang, De’an Tian, Lei Chen, Wen Tang, Vladimir Borzan, Zeljko Krznaric, Miroslava Volfova, Jiri Pumprla, Lucie Veberova, Michal Tichy, Jan Gregar, David Stepek, Jan Ulbrych, Pavel Drastich, Zdenek Papik, Jan Matous, Martin Lukas, Radka Koskova, Jan Fallingborg, Romain Altwegg, Ginette Fotsing, David Laharie, Stephane Nancey, Laurent Peyrin, Stefan Schreiber, Jörg Hoffmann, Ursula Seidler, Lars Fechner, Katrin Arelin, Christel Contzen, Guido Trenn, Thomas Schaum, Katrin Schoettker, Oliver Bachmann, Jens Encke, Michael Ibe, Christian Jakobeit, Marta Varga, Agnes Salamon, Andras Kafony, Gyula Horvat, Zsanett Heringh, Katalin Bezzegh, Ferenc Izbeki, Laszlo Szalai, Robert Schnabel, Jayanta Samanta, Rupa Banerjee, Ganesh Subramanian, Shrikant Mukewar, Naresh Kumar Bansal, Ajit Sood, Ravi Shankar Bagepally, Porika Shravan Kumar, Pankaj Shrimal, Saumin Shah, Nitin Pai, Vineet Ahuja, Ehud Melzer, Nimer Assy, Eran Goldin, Haim Shirin, Arik Segal, Adi Lahat, Simone Saibeni, Francesco Luzza, Silvio Danese, Flavio Caprioli, Maria Laura Annunziata, Massimo Fantini, Koichiro Matsuda, Sho Takagi, Shuji Kanmura, Mitsuhide Goto, Toshifumi Ashida, Nobuaki Nishimata, Kazuhiko Kawakami, Yoh Ishiguro Ishiguro, Atsuo Maemoto Maemoto, Yuji Naito Naito, Hideaki Naoe, Hiroshi Nakase, Tomohiro Iida, Junichi Akiyama, Masao Yoshioka, Yasuhiko Abe, Ken Takeuchi, Mikio Kawai, Fumihito Hirai, Noriyuki Horiki, Sang Hyoung Park, Dong Park, Sang Bum Kang, Min Kyu Jung, Byung Ik Jang, Yoo Jin Lee, SungJae Shin, Tae-oh Kim, Hyun-Soo Kim, Bo-In Lee, Jonghun Lee, Hyo Jong Kim, Chang Hwan Choi, Dongwoo Kim, Chang Soo Eun, Ben Kang, Juris Pokrotnieks, Gediminas Kiudelis, Karina Ramirez, Jose Luis Vega Fonseca, Robert Laheij, Jaroslaw Kierkus, Maria Klopocka, Zbigniew Wylegala, Beata Mroziak, Katarzyna Wojcik, Bartosz Korczowski, Beata Gawdis, Wit Danilkiewicz, Magdalena Olszanecka, Jolanta Krzykowska, Zofia Jamrozik, Malgorzata Duszynska, Agnieszka Ciesiolkiewicz, Malgorzata Baluta, Marek Horynski, Jaroslaw Leszczyszyn, Grzegorz Rozumek, Lukasz Firkowski, Marzena Konopko, Jakub Orleanski, Rafal Filip, Anna Wiechowska, Krzysztof Niezgoda, Radu Mateescu, Eugeniu Craciun, Camelia Chioncel, Marioara Musat, Theodor Alexandru Voiosu, Vasily Trofimov, Oksana Shchukina, Anatoly Pershko, Olga Fedorishina, Galina Chumakova, Igor Bakulin, Marina Osipenko, Denis Nikolin, Olga Barysheva, Olga Alexeeva, Konstantin Zakharov, Irina Khodareva, Alexander Tkachev, Alexey Golovenko, Veronika Popova, Vladimir Kashnikov, Maria Vershinina, Pavel Makarchuk, Ekaterina Valuyskikh, Slobodanka Crevar, Petar Svorcan, Tatjana Radakovic, Tibor Hlavaty, Ivan Bunganic, Miroslav Fedurco, Iveta Kalisova, Jozef Balaz, Lubomir Mihalkanin, Maria Dolores Martin Arranz, Luis De Teresa Parreno, Miquel Sans Cuffi, Alejandro Hernandez Camba, Stephan Brand, Pascal Juillerat, Frank Seibold, Hakan Demirci, Ahmet Tezel, Halis Simsek, Murat Kiyici, Can Gonen, Sadettin Hulagu, Tarkan Karakan, Ayhan Cekin, Engin Altintas, Kadri Guven, Hale Akpinar, Yasemin Ozin, Yusuf Erzin, Mehmet Demir, Tuncer Temel, Tetiana Lohdanidi, Valeriy Ivanov, Olha Ivanishyn, Oleksandr Golovchenko, Oksana Gerasymenko, Svitlana Danyliuk, Vira Vyshyvanyuk, Olena Datsenko, Mykola Stanislavchuk, Yaroslava Rishko, Olga Kyrychenko, Dmytro Donets, Yana Shapovalova, Andriy Yurkiv, Oleksandr Oliinyk, Nataliia Tsarynna, Oleksandr Fediv, Yevgen Poplyonkin, Arthur Kaser, Syed Hoque, Hawys Thomas, Shalini Iyengar, Frederic Newman, Ronald Fogel, Peder Pedersen, Chad Gonzales, Gregory McCord, Michael Galambos, Paul Lamb, Jeffrey Schneider, Bal Raj Bhandari, Michael Weiss, Christine Thai, Michael Shapiro, Ziad Younes, Narayanachar Murali, Jason Hou, Todd Williams, Omer Khalid, Connie Hsu, Christopher Bartalos, Calin Arimie, Syed Mumtaz, Israel Crespo, Renee Marchioni Beery, Anne Tuskey, Ryan Gaible, Alan Cutler, Anita Afzali, Kevin Stuart, Eric Ibegbu, Moises Irizarry-Roman, Kimberly Harris, Kwadwo Agyei, Frances Jones, Aasim Sheikh, Liam Zakko, Erica Cohen, George Duvall, Houssam Al Kharrat, Robert Wohlman, William Holderman, Alan Schulman, Sheldon Lidofsky, Peter Wayne, Luis Victores, Christian Stone, Kenolisa Onwueme, Monika Fischer, Shabana Shahid, Keith Moore, Jeffry Katz, Jeff Bullock, Julien Fahed

**Affiliations:** Department of Medicine, Western University, London, N6A 2L9, Canada; Faculty of Medicine, University of Porto, Porto, 4099-002, Portugal; Immunology, Eli Lilly and Company, Indianapolis, IN, 46285, United States; Faculty of Medicine, KU Leuven, 3000, Belgium; Henry D. Janowitz Division of Gastroenterology, Icahn School of Medicine at Mount Sinai, New York, NY, 10029, United States; Department of Gastroenterology and Hepatology, Kyorin University, Tokyo, 181-8611, Japan; Inflammatory Bowel Disease Centre, Amsterdam University Medical Centers, Amsterdam, 1105 AZ, Netherlands; Department of Pathology and Laboratory Medicine, Sinai Health System, Toronto, ON, M5G 1X5, Canada; Department of Laboratory Medicine and Pathobiology, University of Toronto, Toronto, ON, M5S 1A1, Canada; Immunology, Eli Lilly and Company, Indianapolis, IN, 46285, United States; Immunology, Eli Lilly and Company, Indianapolis, IN, 46285, United States; Immunology, Eli Lilly and Company, Indianapolis, IN, 46285, United States; Immunology, Eli Lilly and Company, Indianapolis, IN, 46285, United States; Immunology, Eli Lilly and Company, Indianapolis, IN, 46285, United States; Head of Inflammatory Bowel Disease and Eosinophilic Esophagitis Division of Gastroenterology & Hepatology, Universitäts Spital Zürich, Zürich, 8006, Switzerland; Division Gastroenterology and Hepatology, Medical University of Vienna, Vienna, 1090, Austria

**Keywords:** mirikizumab, endoscopy, histology, Crohn’s disease

## Abstract

**Background and aims:**

Evaluation of microscopic inflammation is an emerging therapeutic target of Crohn’s disease. The completed VIVID-1 trial evaluated histologic and combined histologic–endoscopic outcomes for mirikizumab relative to placebo and ustekinumab in moderately-to-severely active Crohn’s disease.

**Methods:**

Two specimens from each of five intestinal segments (one ileal, four colonic) were obtained from the edge of ulcers, or the most inflamed mucosa at screening, and weeks (W)12 and 52. Histologic response was defined as the absence of epithelial neutrophils and epithelial damage, erosions and ulceration or ≥50% decrease in either the active Robarts Histopathology Index or the active Global Histologic Disease Activity Score, and was prespecified. Histologic remission was defined as absence of mucosal neutrophils, no epithelial damage, and no erosions and ulcers and was a prespecified endpoint. Post-hoc analyses included subgroup analyses, the proportions of patients achieving combined histologic–endoscopic outcomes, the agreement between histologic outcomes and endoscopic or clinical outcomes, segmental analyses, the assessment of cut-off points for fecal calprotectin, and the association between W12 histologic outcomes and selected W52 outcomes.

**Results:**

At W12, mirikizumab was superior to placebo for histologic response (*P* < .0001) and histologic remission (*P* = .001), with similar rates to ustekinumab (*P* = .58 and *P* = .44, respectively). At W52, mirikizumab-treated patients showed greater histologic response (*P* = .008) compared to ustekinumab, especially in patients who previously failed biologic therapies (*P* = .006). Combined histologic–endoscopic response at W52 was numerically higher for mirikizumab (*P* = .06) and reached statistical significance among biologic-failed patients (*P* = .02); however, combined remission rates were comparable across treatment arms.

**Conclusions:**

Mirikizumab was superior to placebo in achievement of all histology-based endpoints. Mirikizumab showed higher rates compared to ustekinumab for achievement of histologic response at W52. Early histologic response among endoscopic non-responders was associated with 1-year endoscopic outcomes. ClinicalTrials.gov, NCT03926130.

## 1. Introduction

The role of histology in the management of Crohn’s disease (CD) is evolving.^1^^,^^2^ In ulcerative colitis (UC), histology scoring systems were well validated, and multiple observational studies have shown that histologic healing is associated with a better long-term prognosis, defined by decreased rates of relapse, hospitalization, surgery, and colorectal dysplasia,^3–6^ relative to endoscopic healing alone. Similar data in CD are sparse.^5^^,^^7^ Although several studies consistently reported the presence of histologic inflammation in about a quarter of patients^8–10^ with normal endoscopic findings, the precise prognostic importance of histology remains unclear. That is why endoscopy remains the gold standard for the objective evaluation of intestinal inflammation in both clinical trials and clinical practice. However, evidence is evolving, and recent phase 3 CD ­programs^11^ have incorporated histologic evaluation as a secondary efficacy evaluation. In addition, a recent study in postoperative CD^12^ and the nationwide Swedish cohort study highlighted^13^ the long-term impact of histology on relapse and mortality risk in CD.

Inherent challenges with histologic assessment in CD include the lack of standardization for optimal sampling, given the patchy nature of inflammation, heterogeneity in disease location, an incomplete understanding of the microscopic healing process after treatment, and the inability of conventional mucosal biopsies to evaluate transmural inflammation. However, Pai et al. showed that transmural inflammation without microscopic inflammation is unusual; conversely, mucosal inflammation without transmural inflammation is relatively common.^6^ Thus, we speculate that resolution of microscopic mucosal inflammation may reflect healing in deeper bowel layers and could have prognostic importance.

Mirikizumab, a humanized IgG4 monoclonal antibody that specifically binds to the p19 subunit of interleukin (IL)-23, is approved for the treatment of both UC and CD. Mirikizumab has demonstrated efficacy in improving clinical, endoscopic, and biomarker endpoints, along with an acceptable safety profile for patients with moderately-to-severely active CD in the phase 3 VIVID-1 study (NCT03926130).^14^ This trial was unique in that it incorporated comprehensive assessments, allowing for a comparison of the relative efficacy of mirikizumab to both placebo and ustekinumab regarding microscopic inflammation.

## 2. Materials and methods

### 2.1. Study design and participants

VIVID-1 was a phase 3, multicenter, randomized, double-blind, double-dummy, parallel-group, placebo- and active-controlled study (Figure S1). The VIVID-1 study enrolled patients with demonstrated intolerance, inadequate response, or loss of response to conventional and/or biologic therapies. Eligibility requirements have been previously described.^14^ The VIVID-1 protocol was approved by local ethical review boards and ­conducted according to the International Conference on Harmonisation of Good Clinical Practice guidelines and the Declaration of Helsinki.

### 2.2. Study procedures

#### 2.2.1. Endoscopic assessment

The Simple Endoscopic Score for Crohn’s Disease (SES-CD)^15^ was used to evaluate endoscopy videos collected during ileo-colonoscopies at weeks (W) 0,12, and 52. The procedures for endoscopy reading have been previously described.^14^ The central reader scoring is detailed in Table S1. Endoscopies with ≥1 evaluable intestinal segments were included in the analysis. Missing segments were scored as zero as previously reported.^8^

#### 2.2.2. Histologic assessment

Three full colonoscopies with ileal intubation were performed at screening and W12 and W52 (or at the early termination visit).^14^ In each of five segments, two biopsies representative of the greatest mucosal inflammation were collected from the edge of ulcers or erosions if present, or from the most macroscopically involved mucosa if no ulcers or erosions were seen. In the absence of any endoscopic lesions, biopsies were randomly obtained from an uninflamed intestinal segment. The Global Histologic Disease Activity Scoring System (GHAS) and the Robarts Histopathology Index Scoring System (RHI) were used to evaluate both biopsies (Tables S2 and S3); the worst score was the final score for that segment. We scored each segment separately but also combined the four colonic segments to represent global colonic disease activity; all five segments were combined to create an overall score. To achieve histologic response and remission at any time point, the criteria had to be met in all five intestinal segments.

Histologic activity and all histologic scores were determined by a single central reader who was unaware of clinical information, including treatment, assignment, and visit sequence.

Histologic assessment endpoints are defined in Table 1. Those definitions were previously used in the phase 2 mirikizumab CD program (SERENITY)^8^ as advised by the expert group (FM, RP, GDH, NH, WR, BF).

**Table 1.** Descriptions of histologic and endoscopic endpoints and analyses.

Endpoint	Description
**Histologic**	
Histologic response*	Absence of neutrophils in epithelium and absence of epithelial damage, erosions, and ulcerations in all 5 intestinal segments (terminal ileum and 4 colonic segments); neutrophil infiltration of lamina propria is allowed OR Reduction of ≥50% from baseline in the sum of 5 segments with active RHI^a^ or active GHAS^b^
Histologic remission*	Complete absence of mucosal neutrophils (in epithelium and lamina propria), and no epithelial damage, erosions and ulcers in both the active RHI and the active GHAS score (criteria had to be met in all 5 intestinal segments)
Change of RHI score from baseline**	LSM percentage change from baseline is reported using analysis of covariance with treatment, baseline value, prior biologic failure, baseline SES-CD total score (<12, ≥12), and either baseline SF ≥ 7 and/or baseline AP ≥ 2.5.
**Endoscopic**	
Endoscopic response*	≥50% decrease from baseline in SES-CD total score
Endoscopic remission**	SES-CD^c^ of ≤4 for ileal–colonic disease or ≤2 for isolated ileal disease, and no subscore >1 in any individual variable
Segmental endoscopic remission**	Ulcer-free remission in segments with ulcers at baseline
Change of SES-CD score from baseline**	LSM percentage change from baseline is reported using mBOCF carried forward; ANCOVA model including treatment, baseline value, prior biologic failure (yes/no), baseline SES-CD total score (<12, ≥12), and either baseline SF ≥7 and/or baseline AP ≥2·5 (yes or unknown/no)
**Combined**	
Histologic–endoscopic response**	Achieving both, endoscopic response and histologic response
Histologic–endoscopic remission**	Achieving both, endoscopic remission and histologic remission

* Prespecified endpoint based on the PAS population.

** Post-hoc analysis endpoints were evaluated for participants in the PAS with active histologic disease at baseline.

Prespecified endpoints for the PAS population were also analyzed post-hoc for patients with active histologic disease at baseline.

a RHI (Robarts Histopathology Index) is a four-item measurement calculated for all five segments as the sum of all items with multiplication factors (a range of 0–33). Active RHI is defined as the sum of three selected items of RHI.

b GHAS (Global Histology Activity Score) is an eight-item measurement calculated as a sum of the first seven items for all five segments and comprise the last item which was evaluated for all (a range of 0–16). Active GHAS is defined as the sum of four selected items of GHAS.

c SES-CD (Simple Endoscopic Score for Crohn Disease) assesses the size of mucosal ulcers, the ulcerated surface, the endoscopic extension, and the presence of stenosis.

Abbreviations: AP, abdominal pain; LSM, least square means; mBOCF, modified baseline observation carried forward; PAS, primary analysis set; PRO, patient-reported outcome; SF, stool frequency.

### 2.3. Outcomes and statistical analysis

A description of histologic, endoscopic, and clinical outcomes can be found in Table 1. Prespecified histology analyses included the proportion of patients achieving endpoints of histologic response and remission at W12 and W52 across treatment groups. Prespecified analyses were conducted in the primary analysis set (PAS) population, which included patients who had an SES-CD ≥ 7 (or ≥4 for isolated ileal disease) at baseline and received at least one dose of study drug.

Post hoc analyses included subgroup analyses, the proportions of patients achieving combined histologic–endoscopic outcomes, the agreement between histologic outcomes and endoscopic or clinical outcomes, segmental analyses, the assessment of cutoff points for fecal calprotectin (FCP), and the association between W12 histologic outcomes and selected W52 outcomes. Unless otherwise stated, these post-hoc analyses were performed in the PAS population with active histologic disease at baseline, defined as neutrophilic infiltration of the epithelium, lamina propria, or both (a-GHAS > 0 or a-RHI > 0). Subgroup analyses were conducted in patients with and without previous failure to biological therapies (biologic failed and not biologic failed subgroups).

Agreement between baseline disease locations defined by endoscopy or histology in all intestinal segments and for each segment was assessed by Cohen’s *K* coefficient using observed data. Pearson’s correlation analysis was used to evaluate the relationship between histologic disease activity (measured by a-RHI) and endoscopic disease activity (measured by SES-CD) at baseline using observed data. Cohen’s *K* coefficients were calculated to assess the agreement between histologic response and selected endoscopic or clinical outcomes using observed data. The assessment on impact of baseline factors (disease characteristics and concomitant and previous treatments) on W12 histologic response and remission was estimated by using a multivariable logistic regression model. The associations of early endoscopic and histologic response with W52 efficacy outcomes were evaluated by using Fisher’s exact test (binary outcomes) or Kruskal–Wallis test (continuous outcomes).

Segmental analyses were conducted to evaluate histologic outcomes in the terminal ileum, right colon, transverse colon, left colon, and rectum. For analysis of change from baseline RHI score, the PAS population was used. For binary histologic outcomes, patients with active histologic disease at the assessed segment were analyzed. For evaluation of the four colonic segments together, to achieve either histologic response or remission, it had to be met in all four colonic segments. Segmental analyses were also conducted for binary endoscopic outcomes in intestinal segments on patients with segmental SES-CD > 0 at baseline (endoscopic response) and on patients with the presence of ulcers at baseline (endoscopic remission). For analysis of change from baseline in segmental SES-CD score, the PAS population was used.

The optimal cutoffs for FCP at W52 were determined for histologic response, combined histologic and endoscopic response, histologic remission, and combined histologic and endoscopic remission at W52 by maximizing Youden’s index, which balances sensitivity and specificity. Sensitivity, specificity, positive predictive value (PPV), negative predictive value (NPV), and area under the curve (AUC) were calculated. W52 efficacy outcomes among subgroups based on the histologic and endoscopic outcomes at W12 were compared using Fisher’s exact test (binary outcomes) or Kruskal–Wallis test (continuous outcomes).

Subgroups by previous failure to biological therapies were analyzed using an unadjusted Fisher’s exact test. Change from baseline in RHI score and in SES-CD total score per segment was analyzed at W52 by analysis of covariance with the randomization strata described above, and missing data imputed by modified baseline observation carried forward. Unless specified otherwise, missing categorical and continuous data were imputed using non-responder imputation (NRI) and modified baseline observation carried forward (mBOCF), respectively.

Unless otherwise stated, the *P*-value and statistical significance reported are nominal (ie, not adjusted for multiplicity).

## 3. Results

### 3.1. Baseline evaluations

Patient demographics and baseline disease characteristics were generally similar among treatment groups (Table S4). At baseline, 90% (963/1065) of patients in the PAS had active histologic disease, whereas approximately 10% (102/1065) did not. A summary of endoscopic characteristics of patients without active histologic disease at baseline is shown in Table S5; baseline demographics and disease characteristics organized by patients with or without active histologic disease are summarized in Table S6.

A descriptive summary of agreement between histology and endoscopy by disease location by endoscopy and histology data at baseline among all patients (PAS population) is shown in Figure S2. Overall, 59% (627/1065) of patients had the same location of disease defined by both histology and endoscopy. Of 422 patients with isolated colonic disease defined by endoscopy, 26% (111/422) had additional histologic inflammation in the terminal ileum. Of those 26%, 85% had baseline endoscopic activity in the right colon (Table S7). Additional details of the histologic response rates at W12 and W52 for this subgroup are summarized in Figure S3.

### 3.2. Impact of mirikizumab on early histologic outcomes

**Figure 1.**
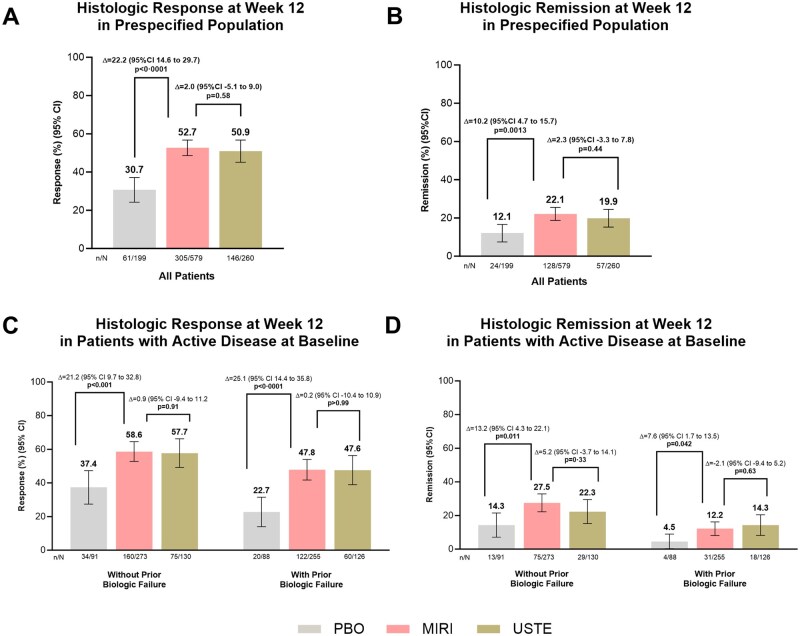
Histologic response (A) and remission (B) at Week 12 among all patients in the prespecified population, and histologic response (C) and remission (D) at Week 12 among patients with active histologic disease at baseline. *All patients were from all randomized patients who received ≥1 dose of allocated treatment with baseline SES-CD ≥ 7 (or ≥4 for isolated ileal disease) (primary analysis set). Analyses in *All patients were prespecified, non-multiplicity-controlled endpoints. Analyses in Endoscopic Response and Clinical Response by PRO are both multiplicity controlled. Abbreviations: PBO, placebo; MIRI, mirikizumab; USTE, ustekinumab.

**Figure 2.**
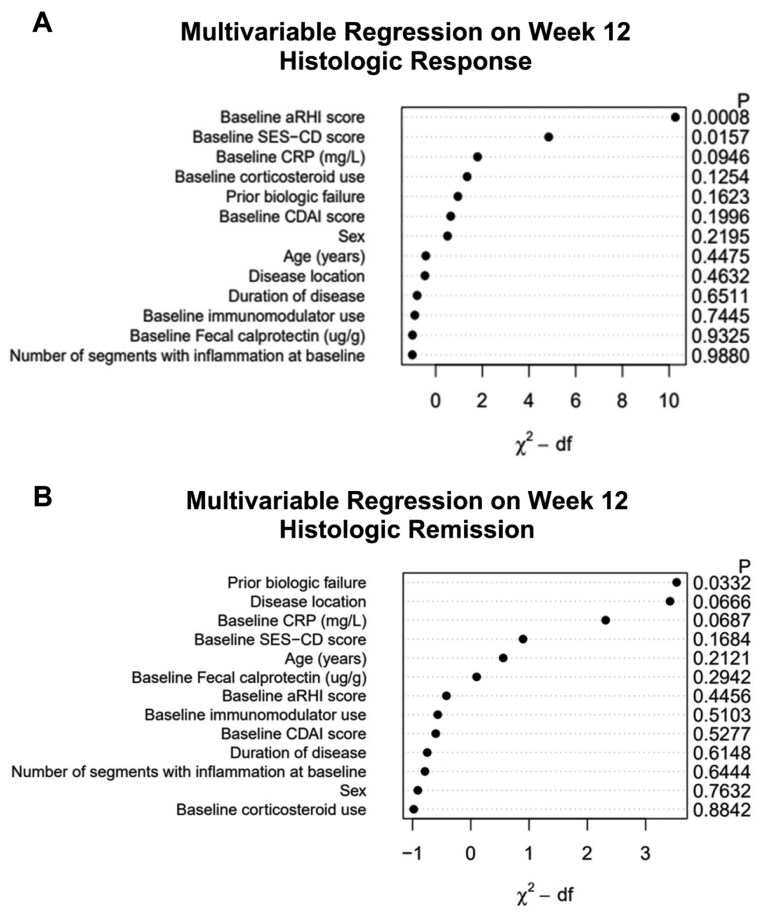
Ranking of predictor importance with Week 12 histologic response (A) and histologic remission (B) based on multivariable logistic regression. The *x*-axis is represented as chi-squared minus degrees of freedom (df), which indicates the strength of the predictor variable’s impact on the outcome and its explanatory power in the model. A larger value of Wald chi-square minus df indicates a higher importance of the predictor, meaning it provides more explanatory power to the model. Corresponding *P*-values <.05 indicate that the predictor variable contributes significantly to the model. Abbreviations: RHI, Robarts Histopathology Index; CRP, C-reactive protein; CDAI, Crohn’s disease Activity Index; SES-CD, Simple Endoscopic Score for Crohn’s Disease.

In all patients, there was a greater proportion in the mirikizumab group than in the placebo group who achieved histologic response (53% vs 31%, *P* < .0001) and remission (22% vs 12%, *P* = .0013) across all intestinal segments at W12 (Figure 1A-B). Mirikizumab and ustekinumab achieved similar rates of early histologic response (53% vs 51%) and remission (22% vs 20%) at W12 (Figure 1A-B). This was similar in patients with active histologic disease at baseline (Figure S4) and did not differ in histologic endpoints depending on prior biologic failure (Figure 1C-D). The congruence between histologic and endoscopic response at W12 was achieved in 62.1% of patients with fair agreement (*K* = 0.27); however, there was no agreement between histologic response and clinical outcomes (Table S8). Multivariable regression analysis showed that only baseline histologic and endoscopic disease severity was associated with early histologic response, while only previous history in biologic failure was significantly associated with histologic remission. Baseline use of corticosteroids or immunomodulators did not have an impact on histologic response or remission (Figure 2; Tables S9 and S10).

### 3.3. Impact of mirikizumab on 1-year histologic outcomes

**Figure 3.**
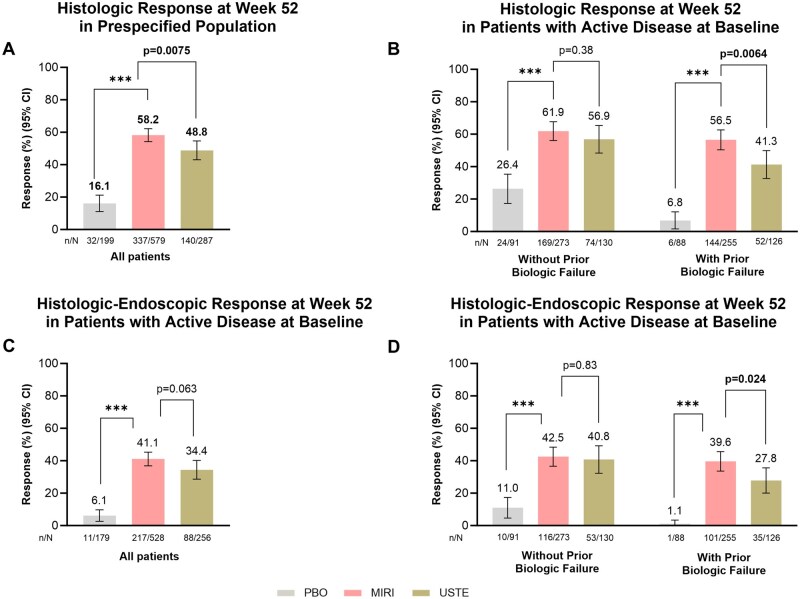
Histologic response among all patients in the prespecified population at Week 52 (A) and combined histologic–endoscopic response at Week 52: (A) histological response all patients, (B) with and without biologic failure, and (C) histologic–endoscopic response for all patients with and without biologic failure who had active disease at baseline. ***P < .001: *P*-values for “All patients” and “All active patients” were from the Cochran–Mantel–Haenszel test adjusting for baseline covariates; Analyses in *Patients with prior biologic failure or *Patients without prior biologic failure” were post-hoc. Participants who were randomized to PBO and switched to MIRI at Week 12 were treated as nonresponders at Week 52. Abbreviations: MIRI, mirikizumab; PBO, placebo; USTE, ustekinumab; n, number of patients in the specified category; N, number of patients in the analysis population.

**Figure 4.**
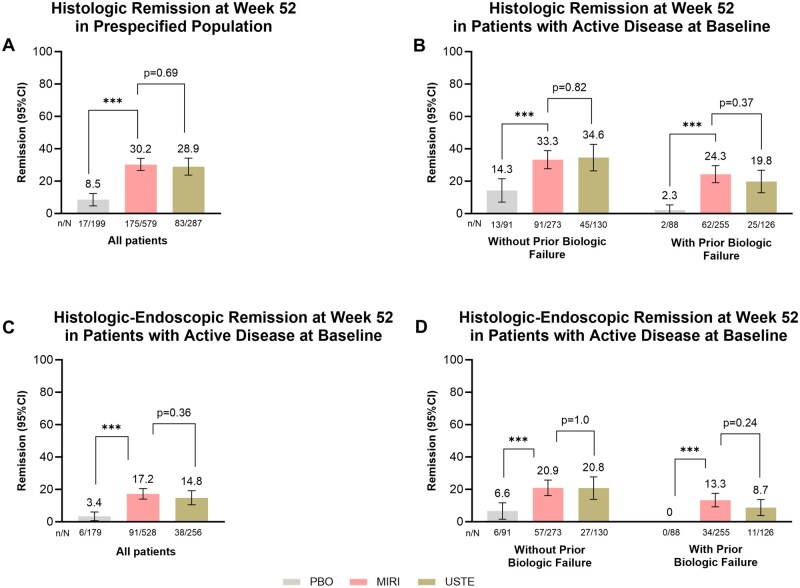
Week 52: (A) Histological remission all patients, (B) histologic remission with and without biologic failure, and (C) histologic–endoscopic remission for all patients with and without biologic failure who had active disease at baseline. **P < .001: *P*-values for “All patients” and “All patients with active disease” were from the Cochran–Mantel–Haenszel test adjusting for baseline covariates. Analyses in *Patients with prior biologic failure or *Patients without prior biologic failure were post-hoc. Participants who were randomized to PBO and switched to MIRI at Week 12 were treated as nonresponders at Week 52. Abbreviations: CI, confidence interval; MIRI, mirikizumab; PBO, placebo; USTE, ustekinumab; n, number of patients in the specified category; N, number of patients in the analysis population.

At W52, a significantly higher proportion of patients achieved histologic response in the mirikizumab group than in the ustekinumab group (58% vs 49%, *P* = .0075) (Figure 3A). This difference was more pronounced among patients who had failed prior biologic therapy (*P* = .0064) (Figure 3B). At W52, a comparable proportion of patients achieved histologic remission in both the mirikizumab and ustekinumab groups (30% vs 29% *P* = .69) (Figure 4A-B). This was also observed in the population of patients with active histologic disease at baseline (29% vs 27%; *P* = .59) (Figure S5).

Among patients with active histologic disease, mirikizumab demonstrated numerically greater combined histologic–endoscopic response rates than ustekinumab in the overall population (41% vs 34%; *P* = .06) (Figure 3C). In the biologic-failed cohort, mirikizumab treatment resulted in a statistically significant improvement over ustekinumab, with 40% reaching combined histologic–endoscopic response versus 28% for ustekinumab (*P* = .024) (Figure 3D). Rates of combined histologic–endoscopic remission were similar between mirikizumab and ustekinumab (*P* = .36), including within the biologic-failed subgroup (*P* = .24) (Figure 4C-D).

Mirikizumab was superior to placebo in achieving composite histologic response (defined as clinical response by patient-reported outcome [PRO] at W12 and histologic response at W52) (*P* < .0001) and composite histologic remission (defined as clinical response by PRO at W12 and histologic remission at W52) at W52 (*P* < .0001) (Figure S6).

The rates of histologic response, histologic remission, and histologic–endoscopic remission did not vary significantly in relation to different disease duration. These rates were also similar to those seen in the general population (Figure S7).

### 3.4. Segmental analysis

**Figure 5.**
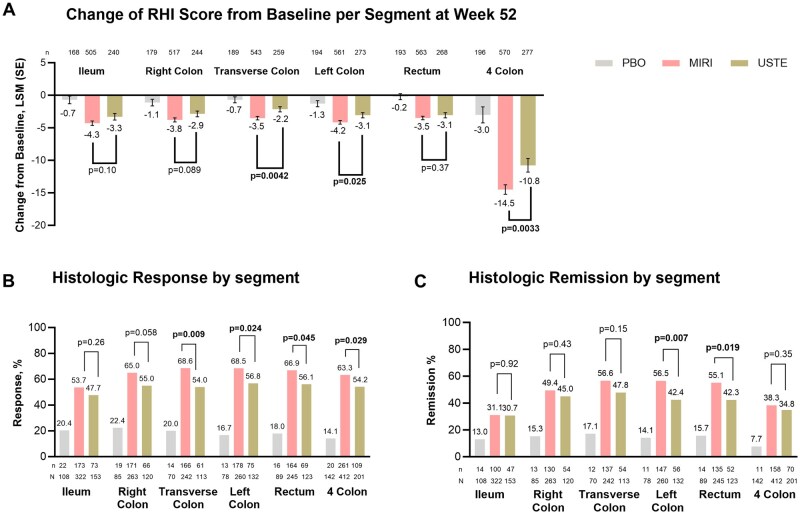
Change in RHI score from baseline per segment (A), histologic response (B), and histologic remission (C) at Week 52. Patients with active histologic disease at baseline were included in this analysis. Abbreviations: MIRI, mirikizumab; PBO, placebo; USTE, ustekinumab; LSM, least square means; n, number of patients in the specified category; N, number of patients in the analysis population; RHI, Robarts Histopathology Index.

Mirikizumab demonstrated a similar effect across all five intestinal segments in reducing histologic inflammation at W52 (measured by the change in RHI from baseline) and achieved statistically significant superiority over placebo. Additionally, it was higher than ustekinumab in the transverse (*P* = .004) and left colon (*P* = .025), as well as when all four colonic segments were analyzed collectively (*P* = .003) (Figure 5A). Regarding segmental endoscopic improvement (measured by the change in SES-CD scores from baseline) at W52, the pattern of differences between arms was similar while only the left colon showed a significant improvement with mirikizumab treatment compared to ustekinumab (*P* = .008) (Figure S8A).

Mirikizumab showed a greater effect than ustekinumab on both histologic response and remission in several colonic segments, notably the transverse colon (response; remission; *P* = .009; *P* = .15), left colon (*P* = .024; *P* = .007), and rectum (*P* = .045; *P* = .019) (Figure 5B-C). Segmental endoscopic response patterns matched those of histologic response, with mirikizumab performing better in the same regions (Figure S8B). Endoscopic remission was statistically superior for mirikizumab only in the left colon (*P* = .014) (Figure S8C). Further comparative data are available in Table S11.

**Figure 6.**
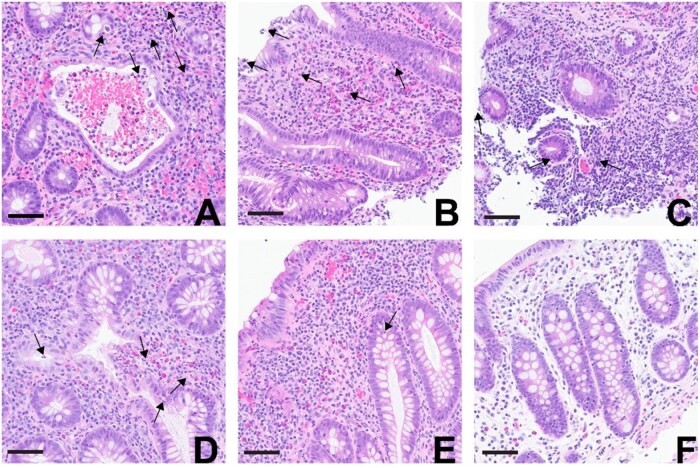
Histologic images. Images are 40× magnification. Scale bar is 60µm. Arrows indicate neutrophils. (A) Active histologic disease at baseline in the transverse colon of a patient randomized to placebo; neutrophil infiltration of epithelium and lamina propria, dense chronic inflammatory infiltrate; crypt abscess in the center of the image. (B) Active histologic disease at week 12 in the transverse colon of a patient treated with placebo; neutrophil infiltration of epithelium and lamina propria with epithelial damage and dense chronic inflammatory infiltrate. (C) Active histologic disease at week 52 in in the transverse colon of a patient treated with placebo; neutrophil infiltration of epithelium and lamina propria, dense chronic inflammatory infiltrate. (D) Active histologic disease at baseline in the transverse colon of a patient randomized to mirikizumab; neutrophil infiltration of epithelium and lamina propria and dense chronic inflammatory infiltrate. (E) Histologic response at week 12 in the transverse colon of a patient treated with mirikizumab; some rare neutrophils in crypts and chronic inflammatory infiltrate. (F) Histologic remission at week 52 in the transverse colon of a patient treated with mirikizumab; no epithelial or lamina propria neutrophils, some rare chronic inflammatory infiltrate.

Histologic images of transverse colon sections taken at baseline and W12 and W52 from patients treated with placebo and mirikizumab are illustrated in Figure 6.

### 3.5. Histologic endpoints and fecal calprotectin

The respective cutoff FCP value in patients who achieved histologic response was 346 µg/g (sensitivity; specificity; 0.721; 0.622), for histologic remission was 158 µg/g (0.691; 0.693), for combined histologic–endoscopic response was 269 µg/g (0.758; 0.622), and for remission was 258 µg/g (0.951; 0.571) (Table S12).

### 3.6. Impact of achieving early histologic and endoscopic endpoints

To further explore the impact of histology beyond endoscopy, we evaluated the impact of achieving W12 endoscopic, histologic, or combined histologic–endoscopic response to the 1-year clinical and endoscopic outcomes (Table 2). Significant differences among the four groups compared (combined histologic–endoscopic, endoscopic-only, histologic-only, and no endoscopic-no histologic response) were observed in achieving endoscopic and histologic outcomes, combined clinical remission and endoscopic response, as well as normalization of FCP. For patients who achieved a W12 endoscopic response, no statistical differences were observed in W52 outcomes between histologic responders and non-responders. For W12 endoscopic non-responders, achieving histologic response had a significant impact on W52 histologic and endoscopic outcomes and FCP change from baseline (Table 2).

**Table 2.** Impact of achieving Week 12 histologic response and endoscopic response to week 52 clinical outcomes.

	Week 12 endoscopic response/non-response						
Outcomes at Week 52	Histologic–endoscopic response (*N* = 131)	Endoscopic response only (*N* = 42)	*P*-value	Histologic response only (*N* = 151)	No-histologic–no endoscopic response (*N* = 204)	*P*-value	Overall *P*-value
**Histologic response, *n* (%)**	99 (75.57%)	30 (71.43%)	.6842	104 (68.87%)	80 (39.22%)	<.0001	<.0001
**Histologic remission, *n* (%)**	61 (46.56%)	14 (33.33%)	.1542	52 (34.44%)	26 (12.75%)	<.0001	<.0001
**Endoscopic response, *n* (%)**	103 (78.63%)	27 (64.29%)	.0680	65 (43.05%)	64 (31.37%)	.0260	<.0001
**Endoscopic remission** ^a^ **, *n* (%)**	76 (58.02%)	18 (42.86%)	.1092	31 (20.53%)	25 (12.25%)	.0395	<.0001
**CDAI remission, *n* (%)**	83 (63.36%)	31 (73.81%)	.2632	82 (54.30%)	95 (46.57%)	.1635	.0013
**Combined CDAI remission + endoscopic response, *n* (%)**	76 (58.02%)	22 (52.38%)	.5925	43 (28.48%)	48 (23.53%)	.3258	<.0001
**CS-free remission, *n* (%)**	79 (60.31%)	29 (69.05%)	.3623	80 (52.98%)	91 (44.61%)	.1330	.0048
**FCP ≤250 µg/g , *n* (%)^a^**	20 (22.47%)	2 (6.90%)	.0968	11 (10.89%)	12 (8.63%)	.6581	.0182
**FCP ≤150 µg/g, *n* (%)^b^**	19 (20.21%)	2 (6.45%)	.0980	11 (9.91%)	9 (6.21%)	.3484	.0088
**Log-transformed FCP change, median (Q1-Q3)^c^**	−2.0 (−3.6, −0.5)	−1.6 (−2.6, 0)	.1410	−1.4 (−2.1, 0)	−0.8 (−1.8, 0)	.0216	<.0001

Data in patients who received mirikuzmab and had histologic disease at baseline.

*N*-values are for the following (histologic–endoscopic response, endoscopic response only, histologic response only, no histologic–no endoscopic response):

a *N* = 89, 29, 101, 139;

b *N* = 94, 31, 111, 145;

c *N* = 103, 36, 123, 160.

Abbreviations: CDAI, Crohn’s disease Activity Index; CS, corticosteroid; FCP, fecal calprotectin.

## 4. Discussion

VIVID-1 is the first phase 3 trial in CD in which intestinal biopsies were systematically collected and reported from five segments at multiple timepoints. The stringent definitions of histologic response and histologic remission used in this study were aligned with the ECCO position statement and expert consensus recommendations.^2^

Mirikizumab showed superiority over placebo in achieving all early and 1-year histologic and combined histologic–endoscopic endpoints in both biologic-failed and not biologic-failed populations. Mirikizumab and ustekinumab demonstrated similar early reductions in histologic inflammation, both substantially exceeding placebo. At W12 of the VIVID-1 study, histologic response rates were consistent with previous results^8^ and recently reported GRAVITI data.^16^

Mirkizumab showed greater improvement in histologic response over time (W12 to W52) than ustekinumab. This aligns with similar numerical trends observed between the two treatments in other VIVID-1 endpoints at W52.^11^ However, the rates for mirikizumab and ustekinumab were similar for the more stringent endpoint of histologic remission at W12 and W52.

Consistent with the results observed in SERENITY^8^ and other trials,^10^^,^^17–19^ there was a moderate concordance between endoscopic and histologic responses at both W12 and W52 (Tables S8 and S11). Notably, the proportion of histologic improvement was consistently higher than that of endoscopic improvement. The multi-variable regression analysis showed that baseline corticosteroids and immunomodulator treatment did not affect relatively high rates of histologic response at W12. Interestingly, among patients who did not achieve endoscopic response at W12, those achieving histologic response had a significantly higher chance of reaching endoscopic response and remission after 1 year of treatment with mirikizumab. Our data show that early histologic improvement predicts later endoscopic improvement. Additionally, histologic and endoscopic findings were consistently discordant across all segments and anatomical locations. When interpreted in the context of the biopsy protocol, these results demonstrate a reproducible and prognostically meaningful pattern of sequential healing that is different from the traditional perception of sequence of healing observed in UC.^20^

To better understand the differences in treatment response of mirikizumab and ustekinumab in achieving endoscopic and histologic outcomes in VIVID-1, a comprehensive segmental analysis was performed to evaluate histologic and endoscopic response and remission across all intestinal segments at W52. The analysis of separate segments showed a greater effect of mirikizumab versus ustekinumab in three colonic segments for both endoscopic and histologic response. Considering the differences, complexity, sequence of healing, and limitations of the scoring systems for endoscopy and histology, the disagreement observed between endoscopic and histologic response rates in VIVID-1, between mirikizumab and ustekinumab, remains to be fully understood. The stenosing component^21^ can contribute significantly to SES-CD and often is irreversible, while current histology scores do not include any measurement of fibrosis. Due to these complexities, histologic evaluation may add value to the assessment of treatment effectiveness in clinical trials.^21^ In addition, this segmental analysis enabled the evaluation of response to treatment among different segments (in particular, terminal ileum and colonic segments) and demonstrated consistent decreases in intestinal inflammation in each of the five segments with mirikizumab therapy.

Although questions remain about the effect of histology on long-term outcomes,^20^ this study found no significant prognostic advantage for early combined histologic–endoscopic response over isolated endoscopic response. However, in endoscopic non-responders, a histologic response indicated potential for later endoscopic improvement or remission. This predictive relationship goes beyond mere methodology. In CD, as ulcers begin to heal, the ulcer margin itself regresses and demonstrates histologic improvement (resolution of epithelial and lamina propria neutrophils) before the ulcer crater itself closes endoscopically. Thus, the biopsied tissue at the ulcer edge can show histologic response while the ulcer remains visible on endoscopy, not because of measurement artifacts but because microscopic healing at the ulcer margin precedes macroscopic crater closure. Therefore, biopsies and histologic evaluation may guide treatment decisions for endoscopic non-responders.

There is significant variability in the methodology for assessing histologic endpoints in the clinical trials that supported several approved therapies in CD.^10^^,^^17–19^^,^^22^^,^^23^ These trials used different definitions of histologic endpoints and different approaches in biopsy sampling. Methodological variability makes these studies difficult to compare. Similar conclusions came from a recent systematic review with meta-analysis.^24^ However, recently disclosed data from GRAVITI,^16^ a treat-through study of subcutaneous guselkumab versus placebo in moderate to severe CD, showed similar rates of histologic endpoints as observed in VIVID-1, using similar methodology and similar histologic endpoint definitions. However, all limitations of indirect comparison need to be acknowledged.

Growing attention to histology in CD was triggered by repeated bidirectional discrepancies between endoscopic and histologic assessments. In the VIVID-1 study, 10% of patients had no active histologic disease despite active endoscopic disease, mirroring earlier mirikizumab results.^8^ The recent GRAVITI study reported an even higher proportion (∼21%) of disagreement between active histologic and endoscopic disease at baseline. Endoscopic and histologic disease locations matched in approximately 60% of VIVID-1 cases with the same trend also seen in SERENITY.^8^ Agreement on disease activity across five intestinal segments and overall was slightly better (Table S13), with a Pearson correlation coefficient of 0.65. Additionally, among patients with isolated colonic disease and an endoscopically normal terminal ileum, 26% still showed histologic inflammation in the ileum, which is consistent with prior reports.^8^^,^^10^^,^^25^ Nearly half of these patients exhibited no endoscopic activity in the ileum, while approximately one-third had ileal segments missing according to all three central endoscopic readers (Table S7). Among the remaining quarter, two agreed on either the absence of activity or the presence of missing segments. One central histology reader, however, confirmed active inflammation within the ileal mucosa among all these patients. The rates of histologic response efficacy at week 52 in this subpopulation across all three treatment arms aligned with those observed in the overall population. These findings indicate that histologic assessment of the terminal ileum may be valuable, as endoscopy alone might not detect inflammation. For cases where all three central readers reported missing ileal segments, it is likely that biopsies were obtained from the ileocecal valve and interpreted as colonic inflammation by central endoscopic reading; nevertheless, central histologic evaluation confirmed active microscopic inflammation within the ileal mucosa. This observation underscores the potential need to reconsider the paradigm for localization of the ileocecal valve in central endoscopic readings.

In a recent prospective cohort,^26^ similar levels of discordance were found between endoscopic remission and histologic activity in both the neo-terminal ileum (24%) and terminal ileum (21%). A retrospective study^12^ found that 42% of cases had histologic inflammation without endoscopic evidence in the neo-terminal ileum. It also showed that histologic activity in a normal-appearing neo-terminal ileum more than doubled the risk of future macroscopic disease recurrence, even among patients on biologic preventive therapy. These results may be a result of sampling error within clinical trials.

The histologic data coupled with complementary positive data from inflammatory biomarkers,^14^ especially FCP, imply that mirikizumab may have a greater anti-inflammatory effect in the intestinal mucosa than ustekinumab. The cutoff values for FCP observed for histologic–endoscopic response (269 µg/g), histologic–endoscopic remission (258 µg/g), and histologic remission (158 µg/g) are aligned with the recent guidelines.^27^ The relatively high threshold observed for histologic–endoscopic remission was not expected. This finding could be explained by the statistical limitation of a very small sample size in this subgroup for the difficult-to-achieve endpoint, and also may be attributed to the limited interpretation of FCP in CD, due to the patchy nature of inflammation and complex scoring.

A key strength of this study is the comprehensive assessment of predefined histologic endpoints across all five intestinal segments with systematically applied methodology in biopsy sampling at baseline, W12, and W52 in a phase 3, double-blind, double dummy placebo and active controlled trial among 1065 patients. Histologic response and remission were prespecified endpoints in VIVID-1, defined in alignment with the ECCO position paper on CD histology,^2^ and centrally read. The VIVID-1 study is the first phase 3 trial that applied recommended stringent histologic criteria versus placebo and the active comparator ustekinumab but also evaluated combined histologic–endoscopic endpoints and comprehensive segmental analysis at W52.

Limitations include the technical challenge of precisely obtaining biopsy specimens during endoscopies, and the histologic scores used in this trial remain to be validated for CD. Despite the expert groups’ recommendation of obtaining the biopsies from the edge of the ulcers, it has still not been proven that this is the best approach, considering that the patchiness of endoscopic and histologic inflammation may differ.^28^ The protocol lacked specific instructions regarding biopsy acquisition for patients with a history of prior bowel resections. Another limitation is that the analyses in the population with active histologic disease were conducted post-hoc. Even though the difference between the PAS population and those with active histologic disease is around 10%, the main results on prespecified and post-hoc results for the same endpoints (histologic response and histologic remission at W12 and W52 for all three arms) were almost identical (Figures 1, 3 and 4; Figures S4 and S5). Finally, this study has not evaluated the relation between mucosal and transmural inflammation as well as the impact on the longer-term outcomes.

VIVID-1 is the first phase 3 trial in CD with systematic collection of histologic biopsies in all five intestinal segments at baseline, W12, and W52. Our dataset supports the idea that histologic evaluation helps identify small bowel inflammation not detected by endoscopy and may provide valuable data influencing potential treatment choices. Our data also imply that histologic response may precede endoscopic response and showed the potential benefit of IL23p19 over IL23p40 inhibition, especially among patients with previous biologic failure. Further analysis from the long-term follow-up of this cohort will be important to demonstrate the impact of histologic endpoints on long-term, clinically meaningful outcomes, such as surgery and hospitalization.

## Supplementary Material

jjag077_Supplementary_Data

## Figures and Tables

**Table:** 

First name	Last name
**Emiliano**	Tron
**Fernanda Dorado**	Dorado
**Lena**	Thin
**Rupert**	Leong
**Hans**	Seltenreich
**Britt**	Christensen
**Anthony**	Croft
**Simon**	Ghaly
**Robert**	Koch
**Walter**	Reinisch
**Sonja**	Heeren
**Peter**	Bossuyt
**Marc**	Ferrante
**Fabio Luiz**	Maximiano
**Ligia Yukie**	Sassaki
**Carolina**	Baia
**Debora**	Poli
**Alexandre**	de Sousa Carlos
**Carlos Fernando**	de Magalhaes Francesconi
**Alexander**	De Sa Rolim
**Julio**	Razera
**Joaquim**	Simoes Neto
**Rodrigo**	Rocco
**Jonathas**	Stifft
**Jonathan**	Soldera
**Genoile**	Silva
**Cintia**	Mendes Clemente
**Sandra**	Di Felice Boratto
**Luciana**	Teixeira de Campos
**Marcelo**	Rodrigues Borba
**Gregory**	Rosenfeld
**Terry**	Ponich
**Allen**	Lim
**Chadwick**	Williams
**Bruce**	Musgrave
**Stephane**	Gauthier
**Wenjia**	Liu
**Chengdang**	Wang
**Hong**	Guo
**Chunxiao**	Chen
**Yan Chen**	Chen
**Qian**	Cao
**Jie**	Zhong
**Min**	Xu
**Yufang**	Wang
**Changqing**	Zheng
**Xiang**	Gao
**Youxiang**	Chen
**Yinglei**	Miao
**Lin**	Wang
**Deliang**	Liu
**Bangmao**	Wang
**Zhengji**	Song
**Xiaowei**	Liu
**Baili**	Chen
**Yi**	Jiang
**Qiang**	Zhan
**Kaiguang**	Zhang
**Hong**	Shen
**Zhaotao**	Li
**Xiaoyan**	Wang
**Mei Wang**	Wang
**De’an**	Tian
**Lei**	Chen
**Wen**	Tang
**Vladimir**	Borzan
**Zeljko**	Krznaric
**Miroslava**	Volfova
**Jiri**	Pumprla
**Lucie**	Veberova
**Michal**	Tichy
**Jan**	Gregar
**David**	Stepek
**Jan**	Ulbrych
**Pavel**	Drastich
**Zdenek**	Papik
**Jan**	Matous
**Martin**	Lukas
**Radka**	Koskova
**Jan**	Fallingborg
**Romain**	Altwegg
**Ginette**	Fotsing
**David**	Laharie
**Stephane**	Nancey
**Laurent**	Peyrin
**Stefan**	Schreiber
**Jörg**	Hoffmann
**Ursula**	Seidler
**Lars**	Fechner
**Katrin**	Arelin
**Christel**	Contzen
**Guido**	Trenn
**Thomas**	Schaum
**Katrin**	Schoettker
**Oliver**	Bachmann
**Jens**	Encke
**Michael**	Ibe
**Christian**	Jakobeit
**Marta**	Varga
**Agnes**	Salamon
**Andras**	Kafony
**Gyula**	Horvat
**Zsanett**	Heringh
**Katalin**	Bezzegh
**Ferenc**	Izbeki
**Laszlo**	Szalai
**Robert**	Schnabel
**Jayanta**	Samanta
**Rupa**	Banerjee
**Ganesh**	Subramanian
**Shrikant**	Mukewar
**Naresh Kumar**	Bansal
**Ajit**	Sood
**Ravi**	Shankar Bagepally
**Porika Shravan**	Kumar
**Pankaj**	Shrimal
**Saumin**	Shah
**Nitin**	Pai
**Vineet**	Ahuja
**Ehud**	Melzer
**Nimer**	Assy
**Eran**	Goldin
**Haim**	Shirin
**Arik**	Segal
**Adi**	Lahat
**Simone**	Saibeni
**Francesco**	Luzza
**Silvio**	Danese
**Flavio**	Caprioli
**Maria Laura**	Annunziata
**Massimo**	Fantini
**Koichiro**	Matsuda
**Sho**	Takagi
**Shuji**	Kanmura
**Mitsuhide**	Goto
**Toshifumi**	Ashida
**Nobuaki**	Nishimata
**Kazuhiko**	Kawakami
**Yoh Ishiguro**	Ishiguro
**Atsuo Maemoto**	Maemoto
**Yuji Naito**	Naito
**Hideaki**	Naoe
**Hiroshi**	Nakase
**Tomohiro**	Iida
**Junichi**	Akiyama
**Masao**	Yoshioka
**Yasuhiko**	Abe
**Ken**	Takeuchi
**Mikio**	Kawai
**Fumihito**	Hirai
**Noriyuki**	Horiki
**Sang Hyoung**	Park
**Dong II**	Park
**Sang Bum**	Kang
**Min Kyu**	Jung
**Byung Ik**	Jang
**Yoo Jin**	Lee
**SungJae**	Shin
**Tae-oh**	Kim
**Hyun-Soo**	Kim
**Bo-In**	Lee
**Jonghun**	Lee
**Hyo Jong**	Kim
**Chang Hwan**	Choi
**Dongwoo**	Kim
**Chang Soo**	Eun
**Ben**	Kang
**Juris**	Pokrotnieks
**Gediminas**	Kiudelis
**Karina**	Ramirez
**Jose Luis**	Vega Fonseca
**Robert**	Laheij
**Jaroslaw**	Kierkus
**Maria**	Klopocka
**Zbigniew**	Wylegala
**Beata**	Mroziak
**Katarzyna**	Wojcik
**Bartosz**	Korczowski
**Beata**	Gawdis
**Wit**	Danilkiewicz
**Magdalena**	Olszanecka
**Jolanta**	Krzykowska
**Zofia**	Jamrozik
**Malgorzata**	Duszynska
**Agnieszka**	Ciesiolkiewicz
**Malgorzata**	Baluta
**Marek**	Horynski
**Jaroslaw**	Leszczyszyn
**Grzegorz**	Rozumek
**Lukasz**	Firkowski
**Marzena**	Konopko
**Jakub**	Orleanski
**Rafal**	Filip
**Anna**	Wiechowska
**Krzysztof**	Niezgoda
**Radu**	Mateescu
**Eugeniu**	Craciun
**Camelia**	Chioncel
**Marioara**	Musat
**Theodor Alexandru**	Voiosu
**Vasily**	Trofimov
**Oksana**	Shchukina
**Anatoly**	Pershko
**Olga**	Fedorishina
**Galina**	Chumakova
**Igor**	Bakulin
**Marina**	Osipenko
**Denis**	Nikolin
**Olga**	Barysheva
**Olga**	Alexeeva
**Konstantin**	Zakharov
**Irina**	Khodareva
**Alexander**	Tkachev
**Alexey**	Golovenko
**Veronika**	Popova
**Vladimir**	Kashnikov
**Maria**	Vershinina
**Pavel**	Makarchuk
**Ekaterina**	Valuyskikh
**Slobodanka**	Crevar
**Petar**	Svorcan
**Tatjana**	Radakovic
**Tibor**	Hlavaty
**Ivan**	Bunganic
**Miroslav**	Fedurco
**Iveta**	Kalisova
**Jozef**	Balaz
**Lubomir**	Mihalkanin
**Maria Dolores**	Martin Arranz
**Luis**	De Teresa Parreno
**Miquel**	Sans Cuffi
**Alejandro**	Hernandez Camba
**Stephan**	Brand
**Pascal**	Juillerat
**Frank**	Seibold
**Hakan**	Demirci
**Ahmet**	Tezel
**Halis**	Simsek
**Murat**	Kiyici
**Can**	Gonen
**Sadettin**	Hulagu
**Tarkan**	Karakan
**Ayhan**	Cekin
**Engin**	Altintas
**Kadri**	Guven
**Hale**	Akpinar
**Yasemin**	Ozin
**Yusuf**	Erzin
**Mehmet**	Demir
**Tuncer**	Temel
**Tetiana**	Lohdanidi
**Valeriy**	Ivanov
**Olha**	Ivanishyn
**Oleksandr**	Golovchenko
**Oksana**	Gerasymenko
**Svitlana**	Danyliuk
**Vira**	Vyshyvanyuk
**Olena**	Datsenko
**Mykola**	Stanislavchuk
**Yaroslava**	Rishko
**Olga**	Kyrychenko
**Dmytro**	Donets
**Yana**	Shapovalova
**Andriy**	Yurkiv
**Oleksandr**	Oliinyk
**Nataliia**	Tsarynna
**Oleksandr**	Fediv
**Yevgen**	Poplyonkin
**Arthur**	Kaser
**Syed**	Hoque
**Hawys**	Thomas
**Shalini**	Iyengar
**Frederic**	Newman
**Ronald**	Fogel
**Peder**	Pedersen
**Chad**	Gonzales
**Gregory**	McCord
**Michael**	Galambos
**Paul**	Lamb
**Jeffrey**	Schneider
**Bal Raj**	Bhandari
**Michael**	Weiss
**Christine**	Thai
**Michael**	Shapiro
**Ziad**	Younes
**Narayanachar**	Murali
**Jason**	Hou
**Todd**	Williams
**Omer**	Khalid
**Connie**	Hsu
**Christopher**	Bartalos
**Calin**	Arimie
**Syed**	Mumtaz
**Israel**	Crespo
**Renee**	Marchioni Beery
**Anne**	Tuskey
**Ryan**	Gaible
**Alan**	Cutler
**Anita**	Afzali
**Kevin**	Stuart
**Eric**	Ibegbu
**Moises**	Irizarry-Roman
**Kimberly**	Harris
**Kwadwo**	Agyei
**Frances**	Jones
**Aasim**	Sheikh
**Liam**	Zakko
**Erica**	Cohen
**George**	Duvall
**Houssam**	Al Kharrat
**Robert**	Wohlman
**William**	Holderman
**Alan**	Schulman
**Sheldon**	Lidofsky
**Peter**	Wayne
**Luis**	Victores
**Christian**	Stone
**Kenolisa**	Onwueme
**Monika**	Fischer
**Shabana**	Shahid
**Keith**	Moore
**Jeffry**	Katz
**Jeff**	Bullock
**Julien**	Fahed

## Data Availability

Eli Lilly and Company provides access to all individual participant data collected during the trial, after anonymization, with the exception of pharmacokinetic or genetic data. Data are available to request 6 months after the indication studied has been approved in the US and EU and after primary publication acceptance, whichever is later. No expiration date of data requests is currently set once data are made available. Access is provided after a proposal has been approved by an independent review committee identified for this purpose and after receiving a signed data sharing agreement. Data and documents, including the study protocol, statistical analysis plan, clinical study report, and blank or annotated case report forms, will be provided in a secure data sharing environment. For details on submitting a request, see the instructions provided at www.vivli.org.

## Notes

**VIVID Study Group investigators** 
