# Supplementary material for: Histologic and combined histologic–endoscopic outcomes with mirikizumab in Crohn’s disease: VIVID-1 trial results
Source: J Crohns Colitis. 2026 Jun 17;20(6):jjag077. doi: 10.1093/ecco-jcc/jjag077 (PMC13275131; doi:10.1093/ecco-jcc/jjag077)
Supplement: jjag077_Supplementary_Data [file jjag077_supplementary_data.docx]

**Supplemental Appendix**

**Histologic and Combined Histologic-Endoscopic Outcomes with Mirikizumab in Crohn’s Disease: VIVID-1 Trial Results**

Table of Contents

[Supplementary Figure 1. VIVID-1 study design and key eligibility criteria. 4](#_Toc226617465)

[Supplementary Figure 2. Patients with baseline active disease by location as determined by histology or endoscopy 5](#_Toc226617466)

[Supplementary Figure 3. Histological Response at Week 12 (A) and 52 (B) in patients with active histologic disease at ileum but without endoscopic disease at ileum at baseline. 6](#_Toc226617468)

[Supplement Figure 4. A) Histologic response and B) histologic remission at week 12 in patients with active histologic disease at baseline. 7](#_Toc226617469)

[Supplement Figure 5. A) Histologic response and B) histologic remission at week 52 in patients with active histologic disease at baseline. 8](#_Toc226617470)

[Supplement Figure 6. A) Composite histologic response, and B) Composite histologic remission at Week 52 in patients with active histologic disease at baseline. 9](#_Toc226617471)

[Supplementary Figure 7. Histological response, histological remission, and histological-endoscopic remission at Week 12 and 52 by disease duration at baseline 11](#_Toc226617472)

[Supplementary Figure 8. Change of SES-CD score from baseline per segment, endoscopic response, and endoscopic remission in 5 intestinal segments at Week 52. 12](#_Toc226617473)

[Supplementary Table 1. Discordance for Readability, SES-CD Score and/or Location. 13](#_Toc226617474)

[Supplementary Table 2. Global Histologic Disease Activity Scoring System (GHAS). 14](#_Toc226617475)

[Supplementary Table 3. Robarts Histopathology Index Scoring System. 15](#_Toc226617476)

[Supplementary Table 4. Baseline demographics and disease characteristics (primary analysis set). 16](#_Toc226617477)

[Supplementary Table 5. Summary of endoscopic characteristics of patients without histologic disease at baseline (primary analysis set). 17](#_Toc226617478)

[Supplementary Table 6. Baseline demographics and disease characteristics by active disease status. 18](#_Toc226617479)

[Supplementary Table 7. Patients with active histologic disease at ileum but without endoscopic disease at ileum at baseline – segmental correlation analysis 19](#_Toc226617480)

[Supplementary Table 8. Association of histologic response with endoscopic response, PRO response, CDAI response, and BU CMI response at Week 12. 20](#_Toc226617481)

[Supplemental Tables 9. Summary Table of Odds Ratio for Week 12 Histologic Response Outcomes based on Multivariable Logistic Regression Model 21](#_Toc226617482)

[Supplemental Tables 10. Summary Table of Odds Ratio for Week 12 Histologic Remission Outcomes based on Multivariable Logistic Regression Model. 22](#_Toc226617483)

[Supplement Table 11. Agreement of endoscopic response and histologic response by segment at Week 52. 23](#_Toc226617484)

[Supplement Table 12. Cut-offs of fecal calprotectin for histologic and combined endoscopic and histologic outcomes^a^ 25](#_Toc226617485)

[Supplement Table 13. Descriptive summary of agreement of endoscopic and histologic disease activities at baseline. 26](#_Toc226617486)

[References 26](#_Toc226617487)

# Supplementary Figure 1. VIVID-1 study design and key eligibility criteria.

**Key Eligibility Criteria**

Age ≥18 to ≤80 years

Moderately to severely active CD as defined as unweighted daily average stool frequency ≥4 and/or unweighted daily average abdominal pain (AP) ≥2 at baseline​

SES-CD ≥7 (or ≥4 for isolated ileal disease) within 21 days before randomization

Inadequate response, loss of response, or intolerance to ≥1 medications including corticosteroid, immunomodulator, or approved biologic therapy for CD​


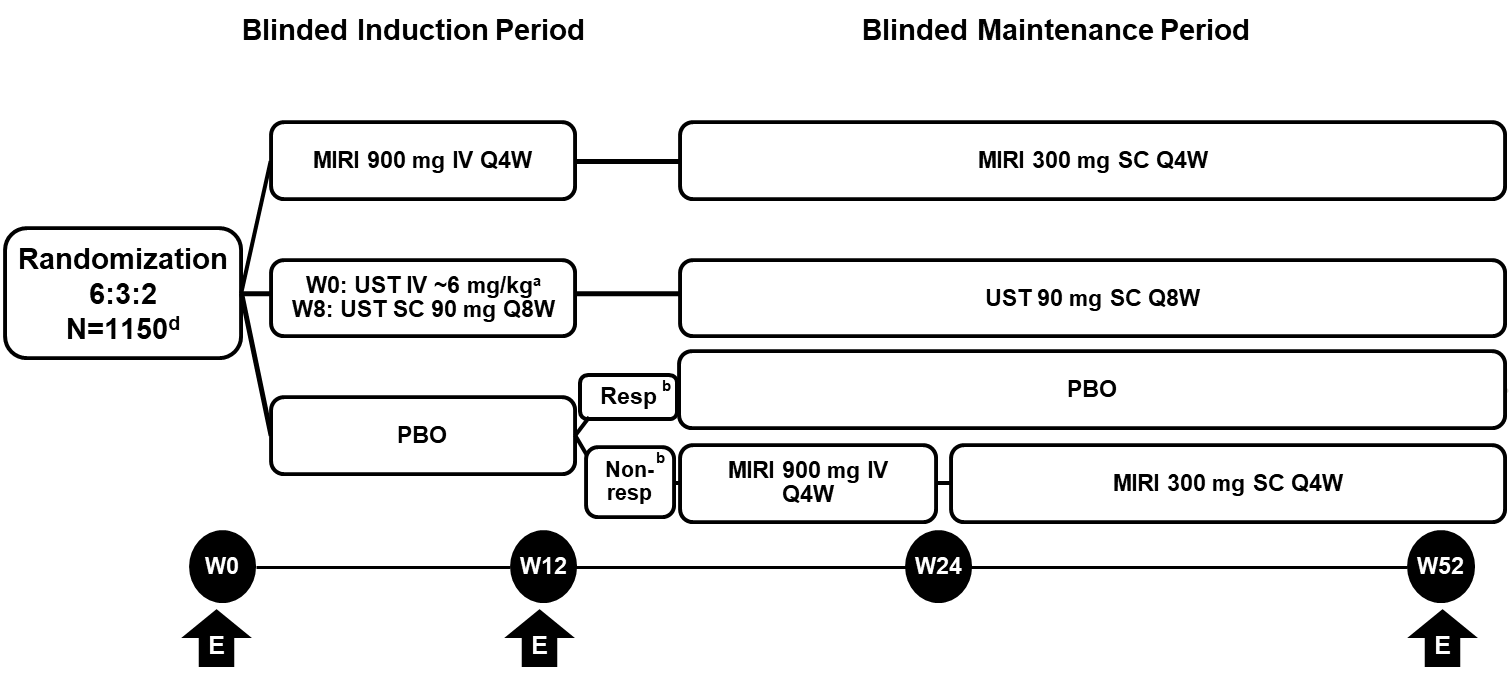


Abbreviations: AP, abdominal pain; CD, Crohn’s disease; E, endoscopy; IV, intravenous; MIRI, mirikizumab; NR, non-responder; PBO, placebo; PRO, Patient Reported Outcome; Q4W, every 4 weeks; Q8W, every 8 weeks; R, responder; SC, subcutaneous; SES-CD, Simple Endoscopic Score for Crohn’s disease; UST, ustekinumab; W, Week.

^a^Single dose; ^b^Responders by PRO at Week 12 of VIVID-1, defined as having achieved ≥30% decrease in loose SF and/or AP, with neither score higher than baseline; ^c^PBO was administered IV and SC from Weeks 8 to 20; otherwise administered IV at Weeks 0 and 4; from Week 24, PBO was administered SC only.

Supplementary Figure 2. Patients with baseline active disease by location as determined by histology or endoscopy.


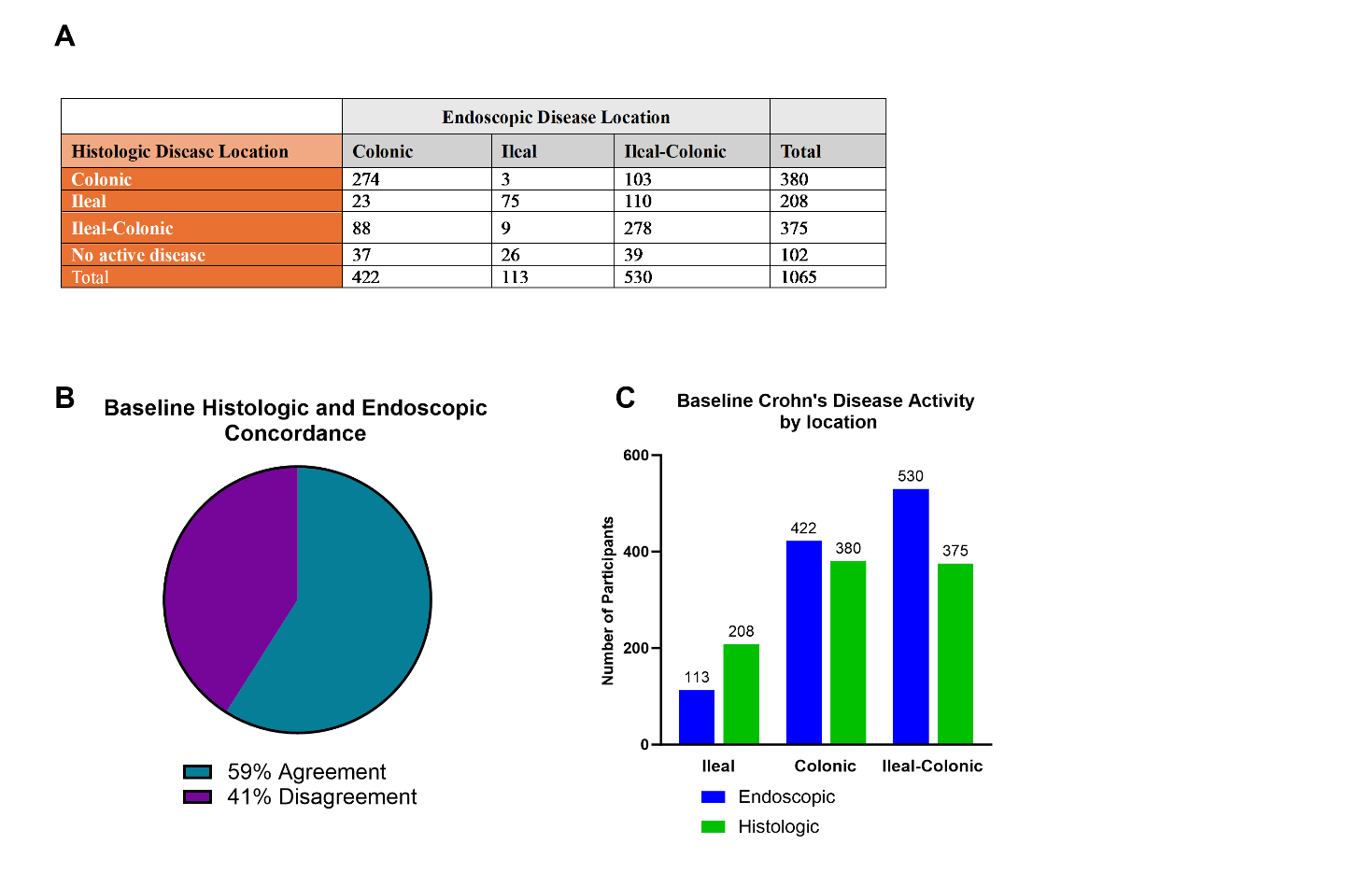
(A) Endoscopic and histologic disease location, (B) Baseline histologic and endoscopic concordance^a^, (C) baseline active disease by location.

^a^ K=0∙38

# Supplementary Figure 3. Histological Response at Week 12 (A) and 52 (B) in patients with active histologic disease at ileum but without endoscopic disease at ileum at baseline.

**
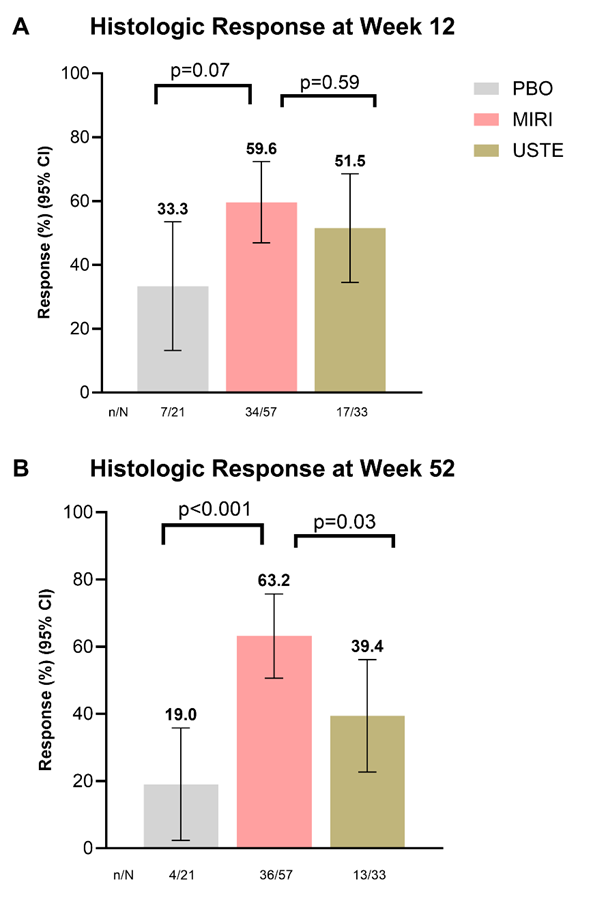
**

All patients from mITT population who have baseline SES-CD≥7 (or≥4 for isolated ileal disease) with active histologic disease at baseline in ileum but without endoscopic disease at ileum at baseline. Histologic response at week 12 in all patients with active histologic disease at baseline were post hoc analyses.

CI=confidence interval; MIRI=mirikizumab; PBO=placebo; n=number of patients in the specified category; N=number of patients in the analysis population; USTE = ustekinumab

# Supplement Figure 4. A) Histologic response and B) histologic remission at week 12 in patients with active histologic disease at baseline.

**
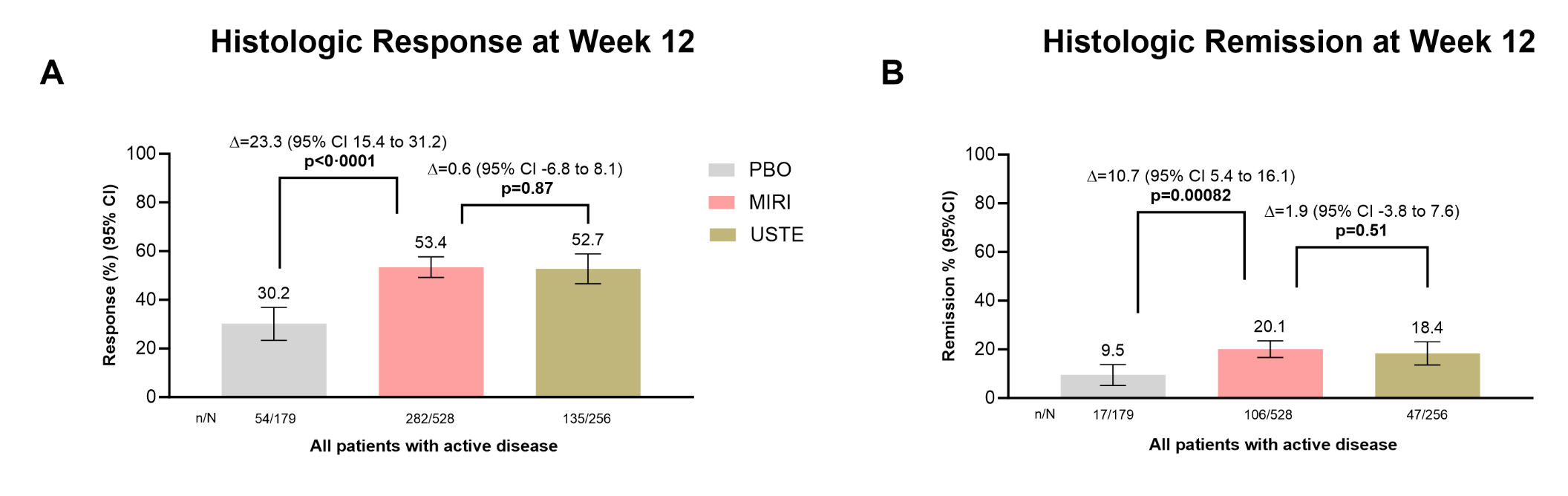
**

All patients from mITT population who have baseline SES-CD≥7 (or≥4 for isolated ileal disease) with active histologic disease at baseline. Histologic response and remission at week 12 in all patients with active histologic disease at baseline were post hoc analyses. Δ is adjusted risk difference.

CI=confidence interval; MIRI=mirikizumab; PBO=placebo; n=number of patients in the specified category; N=number of patients in the analysis population; USTE = ustekinumab

# **
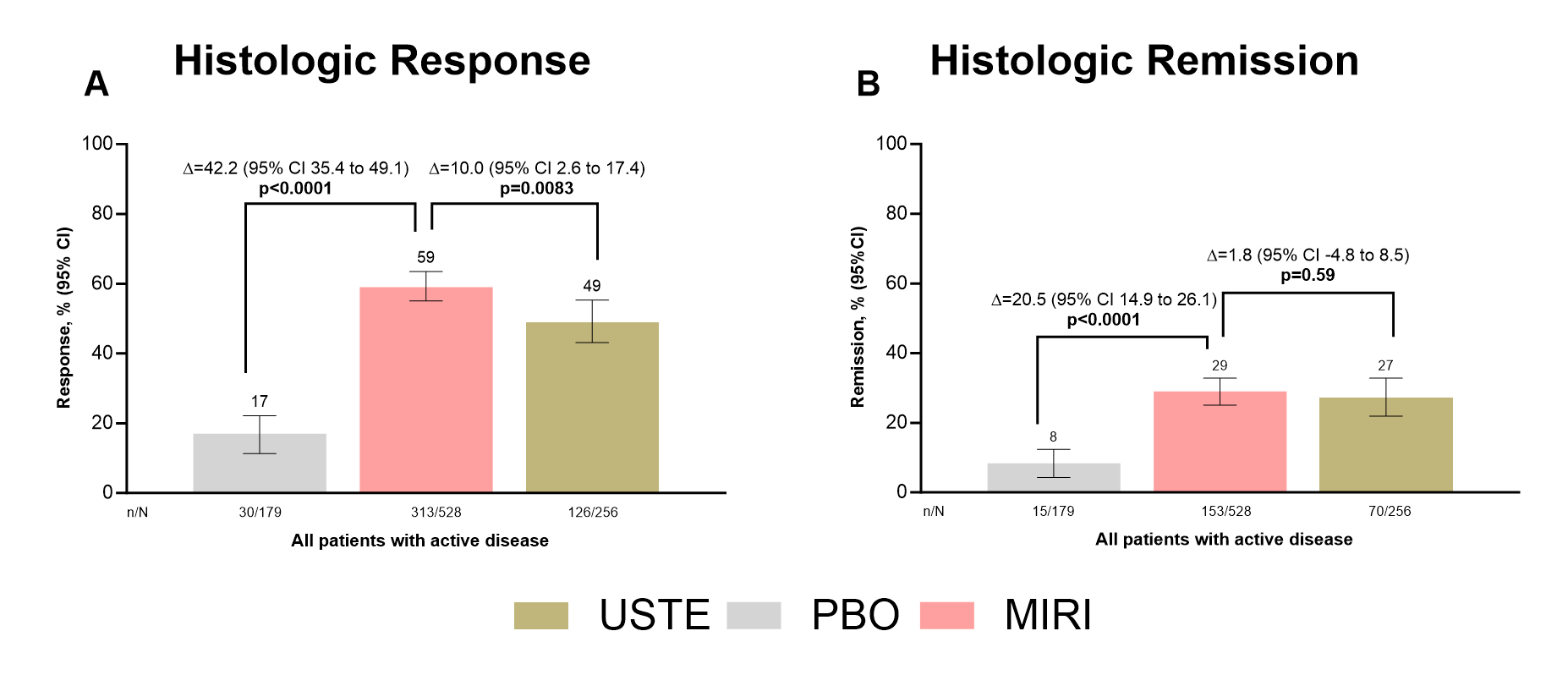
**Supplement Figure 5. A) Histologic response and B) histologic remission at week 52 in patients with active histologic disease at baseline.

All patients from mITT population who have baseline SES-CD≥7 (or ≥4 for isolated ileal disease) with active histologic disease at baseline. Histologic response and remission at week 12 in all patients with active histologic disease at baseline were post hoc analyses. Patients who were randomized to PBO and switched to MIRI at Week 12 were treated as nonresponders at Week 52. Δ is adjusted risk difference.

CI=confidence interval; MIRI=mirikizumab; PBO=placebo; USTE=Ustekinumab; n=number of patients in the specified category; N=number of patients in the analysis population

# Supplement Figure 6. A) Composite histologic response, and B) Composite histologic remission at Week 52 in patients with active histologic disease at baseline.

**
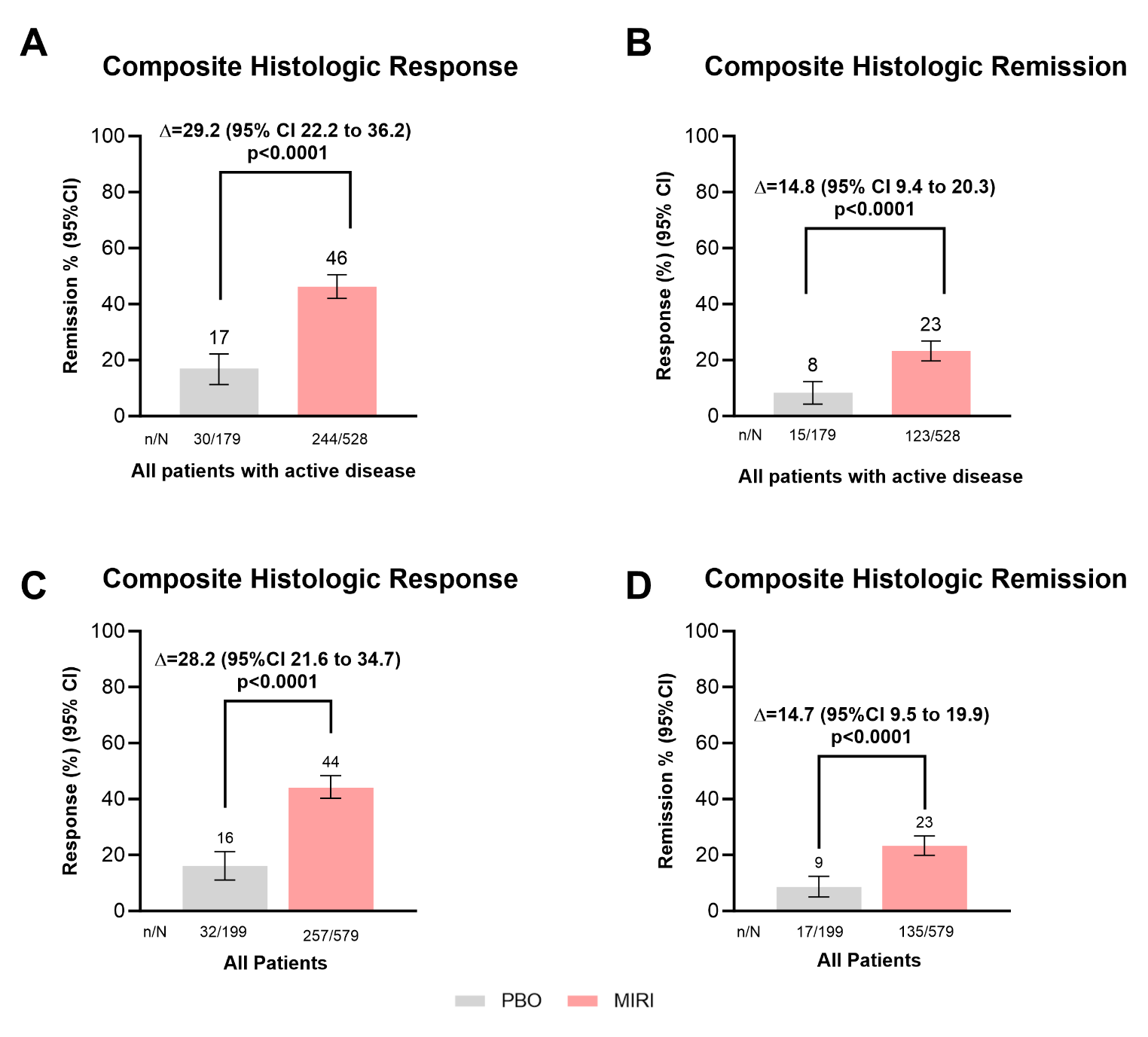
**

All patients from mITT population who have baseline SES-CD≥7 (or ≥4 for isolated ileal disease) with active histologic disease at baseline. Patients who were randomized to PBO and switched to MIRI at Week 12 were treated as nonresponders at Week 52. Histologic response and remission at week 12 in all patients with active histologic disease at baseline were post hoc analyses. Δ is adjusted risk difference.

Composite Histologic Response is defined as Clinical response by PRO at Week 12 and histologic response at Week 52.

Composite Histologic Remission is defined as Clinical response by PRO at Week 12 and histologic remission at Week 52.

Clinical response by PRO is defined as patient reports at least a 30% decrease in SF and/or AP with neither score worse than baseline.

CI=confidence interval; MIRI=mirikizumab; PBO=placebo; PRO= patient-reported outcome; n=number of patients in the specified category; N=number of patients in the analysis population

Supplementary Figure 7. Histological response, histological remission, and histological-endoscopic remission at Week 12 and 52 by disease duration at baseline.


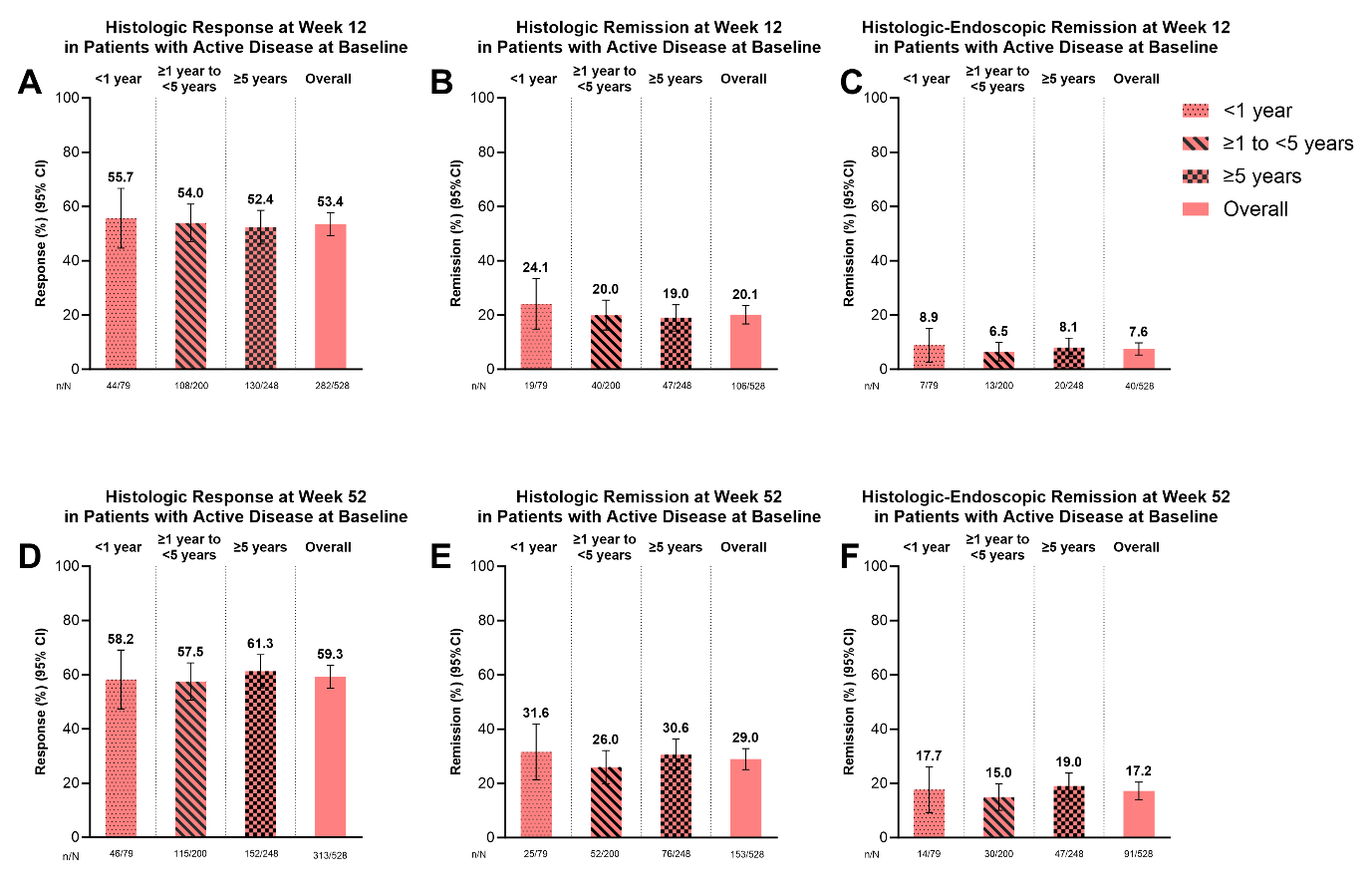


Analyses are post hoc and in patients treated with mirikizumab. Active histologic disease is defined as active GHAS>0 or active RHI>0 at baseline. Abbreviations: CI, confidence interval; n, number of patients in the specified category; N, number of patients in the analysis population.

# Supplementary Figure 8. Change of SES-CD score from baseline per segment, endoscopic response, and endoscopic remission in 5 intestinal segments at Week 52.


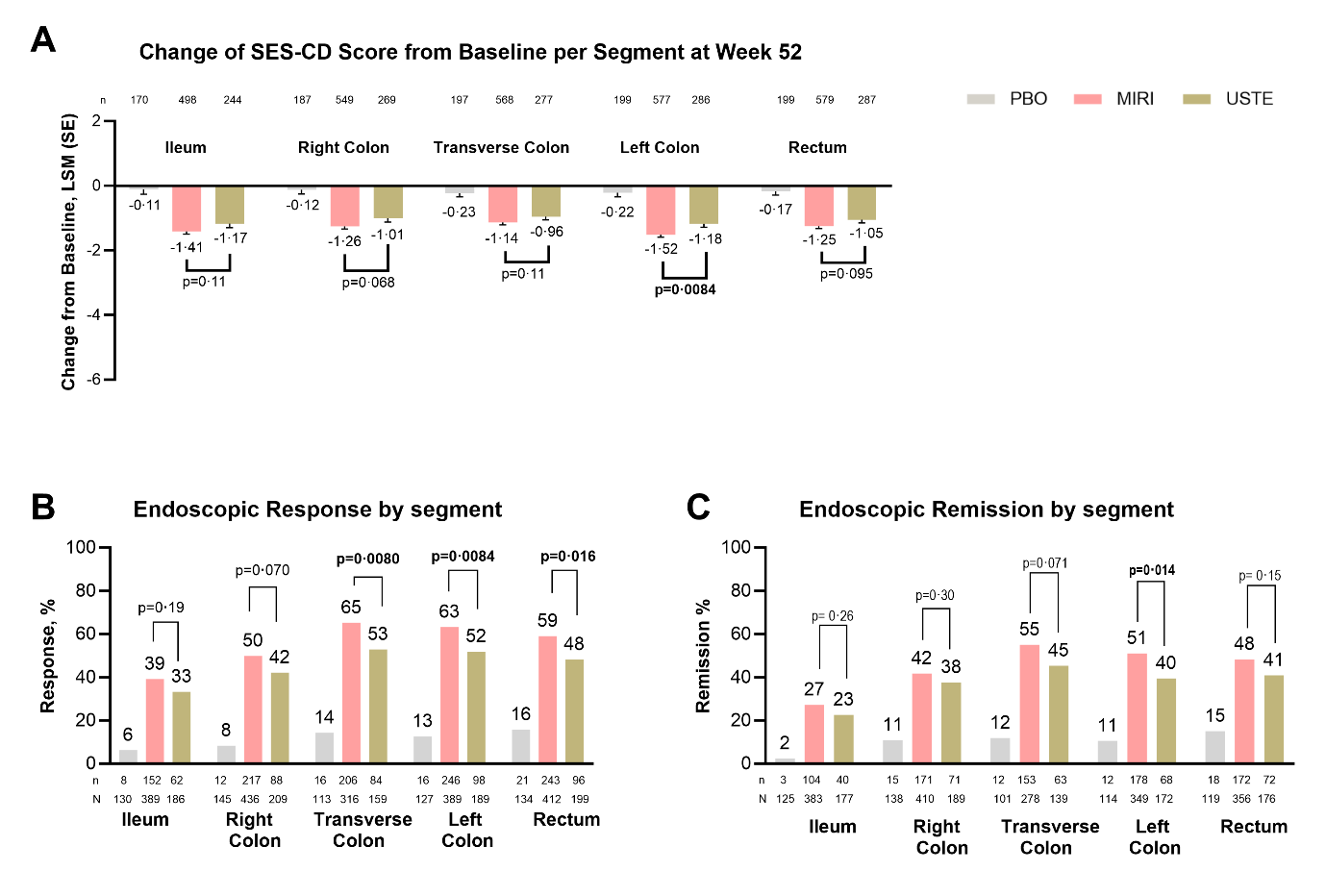


Data includes patients who had a SES-CD ≥7 (or ≥4 for isolated ileal disease) at baseline and received at least one dose of mirikizumab. After Week 12, PBO responders continued receiving PBO; PBO non-responders switched to MIRI, remained in the PBO group, and were imputed as baseline observation carried forward.

(B) Endoscopic response, and (C) endoscopic remission in 5 intestinal segments at week 52.

Patients with segmental SES-CD>0 at baseline (B) and presence of ulcers at baseline (C).

MIRI=mirikizumab; PBO=placebo; USTE=Ustekinumab; SES-CD=Simple Endoscopic Score for Crohn’s Disease; n=number of patients in the specified category; N=number of patients in the analysis population

# Supplementary Table 1. Discordance for Readability, SES-CD Score and/or Location.

| **CR Scoring Results** | **Adjudication** |
| --- | --- |
| Both CR1 and CR2 assign SES-CD score of zero | • No adjudication required, score and location are concordant |
| 1 CR assigns SES-CD score of zero, the other CR assigns SES-CD score above zero | • Adjudication by CR3 required, score and location are discordant |
| Both CR1 and CR2 indicate video is unreadable | • No adjudication required, video will be marked as unreadable, and no SES-CD score will be assigned |
| 1 CR indicates video is unreadable, the other CR indicates video is readable and assigns SES-CD score | • Adjudication by CR3 required readability is discordant  --If CR3 indicates video is unreadable, video will be marked unreadable, and no SES-CD score will be calculated  --If CR3 indicates video is readable, SES-CD score will be determined by calculating the mean of the two available scores |
| Both CR1 and CR2 are concordant for score and location | • No adjudication required |
| CR1 and CR2 are discordant on score and/or disease location | • Adjudication by CR3 required  2 out of 3 CRs agree on disease location:  • Ex. CR1=0, CR2=Ileal colonic, CR3=Ileal colonic →  Final score calculated using all scores, final location is ileal colonic  • Ex. CR1=0, CR2=Ileal, CR3=Ileal → Final score calculated using all scores, final location is ileal  • Ex. CR1=0, CR2=Ileal or ileal colonic, CR3=0 → Final score calculated using all scores, final location is assigned by CR2  All 3 CRs disagree on disease location:  • Ex. CR1=0, CR2=Ileal colonic, CR3=Ileal → Final score calculated using all scores, final location is ileal colonic |
| CR=Central Reader; SES-CD score=Simple Endoscopic Score for Crohn’s Disease  Two CRs, unaware of both treatment assignment and visit sequence, independently scored the SES-CD for each of 5 intestinal segments to determine both initial eligibility and endoscopic disease activity at the previously specified time points. The third CR was used in the case of non-concordance of the first two CRs. In these cases, the final score was the average score of all three readers. | |

# Supplementary Table 2. Global Histologic Disease Activity Scoring System (GHAS).

|  | Item | Score |
| --- | --- | --- |
| 1 | Epithelial damage | 1. Normal 2. Focal pathology 3. Extensive pathology |
| 2 | Architectural changes | 1. Normal 2. Moderately disturbed (<50%) 3. Severely disturbed (>50%) |
| 3 | Infiltration of mononuclear cells in the lamina propria | 1. Normal 2. Moderate increasea 3. Severe increaseb |
| 4 | Infiltration of polymorphonuclear cells in the lamina propria | 1. Normal 2. Moderate increasea 3. Severe increaseb |
| 5 | Polymorphonuclear cells in epithelium | 1. Absent 2. In surface epithelium 3. Cryptitis 4. Crypt abscess |
| 6 | Presence of erosion and/or ulcers | 1. No 2. Yes |
| 7 | Presence of granuloma | 1. No 2. Yes |
| 8 | Number of biopsy specimens affected | 1. None 2. ≤33%   2. 33%-66%  3. >66% |

a Moderate increase, up to twice the number of cells that can normally be expected.

b Severe increase, more than twice the normal number of cells.

The GHAS is a histopathological index consisting of 8 items and calculated as a sum of the first 7 items for all 5 segments and comprise the last item which evaluated for all (a range of 0 to 16). (D’Haens et al. 1998)
The a-GHAS is defined as a sum of Items 1, 4, 5, and 6 (removal of Items 2, 3, 7, and 8) with a worst possible score of 40. The a-GHAS focuses on acute inflammation (Item 3 excluded) (Magro et al. 2024)

The m-GHAS is obtained by adding the chronic inflammation component (infiltration of mononuclear cells in the lamina propria) to the a-GHAS

# Supplementary Table 3. Robarts Histopathology Index Scoring System.

|  | Item | Score | Multiplication Factor |
| --- | --- | --- | --- |
| 1 | Chronic inflammatory infiltrate | 1. No increase 2. Mild but unequivocal increase 3. Moderate increase 4. Marked increase | X1 |
| 2 | Lamina propria neutrophils | 1. None 2. Mild but unequivocal increase 3. Moderate increase 4. Marked increase | X2 |
| 3 | Neutrophils in epithelium | 1. None 2. <5% crypts involved 3. <50% crypts involved 4. >50% crypts involved | X3 |
| 4 | Erosion or ulceration | 1. No erosion, ulceration, or granulation tissue 2. Recovering epithelium + adjacent inflammation 3. Probable erosion-focally stripped 4. Unequivocal erosion 5. Ulcer or granulation tissue | X5 |

The RHI is a histopathologic index consisting of 4 items calculated for all 5 segments, each with score from 0 to 3 (Mosli et al. 2017). The a-RHI is defined as a sum of Items 2, 3, and 4 (removal of Item 1, which focuses on chronic inflammation).

# Supplementary Table 4. Baseline demographics and disease characteristics (primary analysis set).

|  | Mirikizumab  *N* = 579 | Ustekizumab *N* = 287 | Placebo  *N* = 199 |
| --- | --- | --- | --- |
| Age (years), mean (SD) | 36.0 (13.2) | 36.6 (12.7) | 36.3 (12.7) |
| Male, *n* (%) | 332 (57.3) | 137 (47.7) | 118 (59.3) |
| Duration of Crohn’s disease (years), mean (SD) | 7.4 (8.2) | 7.2 (7.7) | 7.8 (7.4) |
| Baseline active histologic disease, *n* (%) | 528 (91.2) | 256 (89.2) | 179 (89.9) |
| RHI, median (IQR) | 29.0 (14.0-54.0) | 30.0 (13.0-54.0) | 29.5 (14.0-48.0) |
| Active RHI, median (IQR) | 25.0 (12.0-47.0) | 26.0 (10.0-47.0) | 26.0 (10.0-42.0) |
| Modified GHAS, median (IQR) | 10.0 (5.0-19.0) | 9.0 (5.0-18.0) | 9.0 (4.5-17.0) |
| Active GHAS, median (IQR) | 7.0 (4.0-14.0) | 7.0 (4.0-15.0) | 7.0 (3.0-13.0) |
| CDAI, mean (SD) | 323.1 (85.8) | 318.5 (93.2) | 318.9 (86.2) |
| SF daily average, mean (SD) | 5.7 (3.0) | 5.7 (2.9) | 5.8 (3.2) |
| AP daily average, mean (SD) | 2.1 (0.6) | 2.1 (0.6) | 2.1 (0.6) |
| SES-CD Total Score, mean (SD) | 13.5 (6.6) | 13.9 (6.6) | 13.1 (6.0) |
| CRP (mg/L), median (IQR) | 8.5 (0.1-205) | 8.9 (0.1-314) | 7.6 (0.1-207) |
| Fecal calprotectin (mg/kg), median (IQR) | 1315 (15-31680) | 1489 (15-31680) | 1161 (15-21164) |
| Endoscopic disease location, *n* (%) |  |  |  |
| Ileum only | 65 (11.2) | 29 (10.1) | 19 (9.5) |
| Colon only | 225 (38.9) | 120 (41.8) | 77 (38.7) |
| Ileum and colon | 289 (49.9) | 138 (48.1) | 103 (51.8) |
| Corticosteroid use, *n* (%) | 177 (30.6) | 90 (31.4) | 58 (29.1) |
| Immunomodulator use, *n* (%) | 146 (25.2) | 87 (30.3) | 58 (29.1) |
| Prior biologic failure, *n* (%) | 281 (48.5) | 139 (48.4) | 97 (48.7) |
| Prior anti-TNF failure, *n* (%) | 265 (45.8) | 133 (46.3) | 89 (44.7) |
| Prior anti-integrin failure, *n* (%) | 68 (11.7) | 31 (10.8) | 24 (12.1) |
| Number of failed biologics, *n* (%) |  |  |  |
| None | 298 (51.5) | 148 (51.6) | 102 (51.3) |
| 1 | 175 (30.2) | 91 (31.7) | 66 (33.2) |
| 2 | 82 (14.2) | 42 (14.6) | 25 (12.6) |
| ≥3 | 24 (4.1) | 6 (2.1) | 6 (3.0) |

Abbreviations: AP, abdominal pain; CD, Crohn’s disease; CDAI, Crohn’s Disease Activity Index; CRP, C-reactive protein; GHAS, Global Histologic Disease Activity Score; IQR, interquartile range; *N*, number of patients in the analysis population; *n*, number of patients in the specified category; RHI, Robarts Histopathology Index; SES-CD, Simple Endoscopic Score for Crohn’s disease; SF, stool frequency; TNF, tumor necrosis factor.

Data include the primary analysis set, which includes all randomized patients who received ≥1 dose of allocated treatment with baseline SES-CD ≥7 (or ≥4 for isolated ileal disease).

# Supplementary Table 5. Summary of endoscopic characteristics of patients without histologic disease at baseline (primary analysis set).

| **Patients without histologic disease**  **at baseline*, *N* = 102** | **Endoscopic disease location** | | |
| --- | --- | --- | --- |
|  | **Colonic**  ***N* = 37** | **Ileal**  ***N* = 26** | **Ileal-colonic**  ***N* = 39** |
| **SES-CD total score** |  |  |  |
| Mean (SD) | 11.59 (5.55) | 5.79 (1.71) | 11.94 (4.62) |
| Median (min, max) | 9.00 (7.0, 25.0) | 5.33 (4.0, 10.0) | 10.50 (7.0, 23.0) |

Abbreviations: SES-CD, Simple Endoscopic Score for Crohn’s disease; N, number of patients in the analysis population; max, maximum; min, minimum.

*Among patients without histologic disease at baseline, patients who had colonic and ileal-colonic disease locations had similar mean SES-CD total scores at baseline, whereas patients with ileal only location had lower mean SES-CD total score and low SES-CD/endoscopic activity.

# Supplementary Table 6. Baseline demographics and disease characteristics by active disease status.

|  | **With Active Disease (N=963)** | **Without Active Disease**  **(N=102)** |
| --- | --- | --- |
| Male, n (%) | 525 (54.5%) | 62 (60.8%) |
| Duration of Crohn’s disease (years), mean (SD) | 7.4 (7.87) | 7.3 (8.51) |
| CDAI, mean (SD) | 321.5 (89.28) | 316.8 (73.80) |
| SF daily average, mean (SD) | 5.8 (3.06) | 5.1 (2.29) |
| AP daily average, mean (SD) | 2.1 (0.60) | 2.2 (0.58) |
| SES-CD Total Score, mean (SD) | 13.9 (6.52) | 10.2 (5.16) |
| CRP (mg/L), median (IQR) | 8.6 (3.1-24.0) | 7.0 (2.7-19.8) |
| Fecal calprotectin (mg/kg), median (IQR) | 1363 (503-2645.5) | 858 (150-2423) |
| Endoscopic disease location, *n* (%) | | |
| Ileum only | 87 (9.0%) | 26 (25.5%) |
| Colon only | 385 (40.0%) | 37 (36.3%) |
| Ileum and colon | 491 (51.0%) | 39 (38.2%) |
| Corticosteroid use, *n* (%) | 296 (30.7%) | 29 (28.4%) |
| Immunomodulator use, *n* (%) | 272 (28.2%) | 19 (18.6%) |
| Prior biologic failure, *n* (%) | 469 (48.7%) | 48 (47.1%) |
| Prior anti-TNF failure, *n* (%) | 440 (45.7%) | 47 (46.1%) |
| Prior anti-integrin failure, *n* (%) | 116 (12.0%) | 7 (6.9%) |
| Abbreviations: AP, abdominal pain; CD, Crohn’s disease; CDAI, Crohn’s Disease Activity Index; CRP, C-reactive protein; IQR, interquartile range; *N*, number of patients in the analysis population; *n*, number of patients in the specified category; SES-CD, Simple Endoscopic Score for Crohn’s disease; SF, stool frequency; TNF, tumor necrosis factor.  Active Histologic disease is defined as active GHAS>0 or active RHI>0 at baseline. | | |

# Supplementary Table 7. Patients with active histologic disease at ileum but without endoscopic disease at ileum at baseline – segmental correlation analysis

| Active histologic disease (a GHAS >0 or aRHI>0) in **ileum** in **isolated colonic disease** (**L2**) by endoscopy N= 111 | | Endoscopic activity (SES CD >0) In Colonic Segments | | | |
| --- | --- | --- | --- | --- | --- |
| Ileum % (n/N) | | Right Colon  % (n/N) | Traverse Colon  % (n/N) | Left Colon  % (n/N) | Rectum  % (n/N) |
| All 3 CRs agreed on SES-CD=0 | **45**(50/111)¥ | **85** (91/107) | **64** (70/109) | **74** (82/111) | **77** (85/111) |
| 2 CRs agreed SES-CD=0 or ileal segment is missing* | **24** (27/111)¥ |  |  |  |  |
| All 3 CRs agreed ileal segment missing | **31** (34/111)# |  |  |  |  |

*Disease location was defined by the agreement of at least 2 CRs

¥ Of the 77 patients with non-missing baseline ileum SES-CD, the baseline active RHI mean at ileum was 10.0

# Of the 34 patients with missing baseline ileum SES-CD, the baseline active RHI mean at ileum was 16.5

# Supplementary Table 8. Association of histologic response with endoscopic response, PRO response, CDAI response, and BU CMI response at Week 12.

|  | **Week 12 Histologic Response** | | |  |
| --- | --- | --- | --- | --- |
|  | **Yes** | **No** | **Total** | **K coefficient (95% CI)** |
| **Week 12 Endoscopic response** |  |  |  |  |
| Response | 132 (29.9) | 39 (8.8) | 171 (38.8) | 0.27 (0.19, 0.35) |
| No Response | 128 (29.0) | 142 (32.2) | 270 (61.2) |  |
| Total | 260 (59.0) | 181 (41.0) | 441 |  |
|  |  |  |  |  |
| **Week 12 Clinical response by PRO** |  |  |  |  |
| Response | 221 (46.4) | 138 (29.0) | 359 (75.4) | 0.077 (-0.0079, 0.16) |
| No Response | 61 (12.8) | 56 (11.8) | 117 (24.6) |  |
| Total | 282 (59.2) | 194 (40.8) | 476 |  |
|  |  |  |  |  |
| **Week 12 Clinical response by CDAI** |  |  |  |  |
| Response | 194 (41.0) | 122 (25.8) | 316 (66.8) | 0.063 (-0.027, 0.15) |
| No Response | 86 (18.2) | 71 (15.0) | 157 (33.2) |  |
| Total | 280 (59.2) | 193 (40.8) | 473 |  |
|  |  |  |  |  |
| **Week 12 Bowel Urgency CMI** |  |  |  |  |
| Response | 128 (26.8) | 73 (15.3) | 201 (42.1) | 0.073 (-0.011, 0.16) |
| No Response | 155 (32.4) | 122 (25.5) | 277 (58.0) |  |
| Total | 283 (59.2) | 195 (40.8) | 478 |  |

Values are n (%)

Population includes patients who received mirikizumab and with active histologic disease at baseline. The agreement of endoscopic response and histologic response is fair. The agreement of PRO response, CDAI response, and BU CMI vs histologic response is none to slight.

Clinical response by Crohn’s Disease Activity Index (CDAI) is defined as a reduction in CDAI score by ≥100 points compared to baseline and/or being in clinical remission by CDAI.

Bowel Urgency Clinically meaningful improvement (CMI) is defined as a ≥3 point improvement in the Urgency Numeric Rating Scale.

Clinical response by PRO is defined as patient reports at least a 30% decrease in SF and/or AP with neither score worse than baseline.

Cohen suggested the Kappa result be interpreted as follows: values ≤ 0 as indicating no agreement and 0.01–0.20 as none to slight, 0.21–0.40 as fair, 0.41– 0.60 as moderate, 0.61–0.80 as substantial, and 0.81–1.00 as almost perfect agreement. (Cohen 1960; McHugh 2012).

BU=bowel urgency; CI=confidence intervals; CDAI=Crohn’s disease Activity Index; CMI=clinically meaningful improvement; PRO=patient reported outcome

Supplemental Tables 9. Summary Table of Odds Ratio for Week 12 Histologic Response Outcomes based on Multivariable Logistic Regression Model.

| **Variable** | **OR (95% CI)** |
| --- | --- |
| Baseline CDAI score | 1.2 (0.91-1.58) |
| Baseline SES-CD score | 0.57 (0.36-0.9) |
| Baseline CRP (mg/L) | 0.85 (0.7-1.03) |
| Baseline fecal calprotectin (ug/g) | 0.99 (0.87-1.13) |
| Baseline age (years) | 1.14 (0.81-1.62) |
| Duration of disease (years) | 0.95 (0.76-1.18) |
| Number of segments with inflammation at baseline | 1 (0.62-1.63) |
| Baseline aRHI score | 1.93 (1.31-2.83) |
| Sex – F:M | 1.3 (0.86-1.97) |
| Disease location – Colonic:Ileal-Colonic | 1.32 (0.84-2.07) |
| Disease location – Ileal:Ileal-Colonic | 1.09 (0.47-2.53) |
| Baseline corticosteroid use – Y:N | 1.42 (0.91-2.23) |
| Baseline immunomodulator use – Y:N | 0.93 (0.59-1.46) |
| Prior biologic failure – Yes:No | 0.74 (0.49-1.13) |
| Abbreviations: OR = odds ratio; Miri = mirikizumab; aRHI = active Robarts Histopathology Index;  DSI-CD = Disease Severity Index-Crohn’s Disease; SES-CD = Simple Endoscopic Score for Crohn’s Disease;  CDAI = Crohn’s Disease Activity Index; CRP = C-reactive protein | |

# Supplemental Tables 10. Summary Table of Odds Ratio for Week 12 Histologic Remission Outcomes based on Multivariable Logistic Regression Model.

| **Variable** | **OR (95% CI)** |
| --- | --- |
| Baseline CDAI score | 0.89 (0.63-1.27) |
| Baseline SES-CD score | 0.64 (0.34-1.21) |
| Baseline CRP (mg/L) | 0.72 (0.5-1.03) |
| Baseline fecal calprotectin (ug/g) | 1.09 (0.93-1.28) |
| Baseline age (years) | 1.3 (0.86-1.97) |
| Duration of disease (years) | 0.94 (0.72-1.21) |
| Number of segments with inflammation at baseline | 1.15 (0.64-2.07) |
| Baseline aRHI score | 0.82 (0.49-1.36) |
| Sex – F:M | 1.08 (0.64-1.83) |
| Disease location – Colonic:Ileal-Colonic | 1.95 (1.1-3.43) |
| Disease location – Ileal:Ileal-Colonic | 1.81 (0.7-4.7) |
| Baseline corticosteroid use – Y:N | 1.04 (0.6-1.81) |
| Baseline immunomodulator use – Y:N | 0.82 (0.46-1.48) |
| Prior biologic failure – Yes:No | 0.55 (0.32-0.95) |
| Abbreviations: OR = odds ratio; Miri = mirikizumab; aRHI = active Robarts Histopathology Index;  DSI-CD = Disease Severity Index-Crohn’s Disease; SES-CD = Simple Endoscopic Score for Crohn’s Disease;  CDAI = Crohn’s Disease Activity Index; CRP = C-reactive protein | |

# Supplement Table 11. Agreement of endoscopic response and histologic response by segment at Week 52.

|  | **Mirikizumab** | | | |
| --- | --- | --- | --- | --- |
|  | **Week 52 Histologic Response** | | | |
|  | **Yes** | **No** | **Total** | **K Coefficient**  **(95% CI)** |
| **Week 52 Endoscopic Response** |  |  |  |  |
| ***Ileum*** |  |  |  |  |
| **Yes** | 83 (40.49) | 9 (4.39) | 92 (44.88) | 0.33  (0.22-0.44) |
| **No** | 63 (30.73) | 50 (24.39) | 113 (55.12) |  |
| **Presence of Narrowing** |  |  |  |  |
| **Yes** | 26 (41.27) |  |  |  |
| **No** | 37 (58.73) |  |  |  |
| **Total** | 146 (71.22) | 59 (28.78) | 205 |  |
|  |  |  |  |  |
| ***Colon, Right*** |  |  |  |  |
| **Yes** | 113 (60.43) | 12 (6.42) | 125 (66.84) | 0.24  (0.10-0.38) |
| **No** | 43 (22.99) | 19 (10.16) | 62 (33.16) |  |
| **Presence of Narrowing** |  |  |  |  |
| **Yes** | 31 (72.09) |  |  |  |
| **No** | 12 (27.91) |  |  |  |
| **Total** | 156 (83.42) | 31 (16.58) | 187 |  |
|  |  |  |  |  |
| ***Colon, Transverse*** |  |  |  |  |
| **Yes** | 122 (73.49) | 14 (8.43) | 136 (81.93) | 0.15  (-0.03-0.33) |
| **No** | 23 (13.86) | 7 (4.22) | 30 (18.07) |  |
| **Presence of Narrowing** |  |  |  |  |
| **Yes** | 4 (17.39) |  |  |  |
| **No** | 19 (82.61) |  |  |  |
| **Total** | 145 (87.35) | 21 (12.65) | 166 |  |
|  |  |  |  |  |
| ***Colon, Left*** |  |  |  |  |
| **Yes** | 133 (69.63) | 8 (4.19) | 141 (73.82) | 0.38  (0.23-0.53) |
| **No** | 31 (16.23) | 19 (9.95) | 50 (26.18) |  |
| **Presence of Narrowing** |  |  |  |  |
| **Yes** | 9 (29.03) |  |  |  |
| **No** | 22 (70.97) |  |  |  |
| **Total** | 164 (85.86) | 27 (14.14) | 191 |  |
|  |  |  |  |  |
| ***Rectum*** |  |  |  |  |
| **Yes** | 115 (61.83) | 7 (3.76) | 122 (65.59) | 0.36  (0.23-0.50) |
| **No** | 40 (21.51) | 24 (12.90) | 64 (34.41) |  |
| **Presence of Narrowing** |  |  |  |  |
| **Yes** | 5 (12.50) |  |  |  |
| **No** | 35 (87.50) |  |  |  |
| **Total** | 155 (83.33) | 31 (16.67) | 186 |  |
|  |  |  |  |  |
| ***All 5 Segments*** |  |  |  |  |
| **Yes** | 217 (52.16) | 30 (7.21) | 247 (59.38) | 0.35 (0.27 – 0.44) |
| **No** | 92 (22.12) | 77 (18.51) | 169 (40.63) |  |
| **Total** | 309 (74.28) | 107 (25.72) | 416 |  |

For patients randomized to mirikizumab: There is a fair agreement between histologic and endoscopic response in ileum, right colon, left colon, and rectum. The agreement is none-to-slight in transverse colon. The majority of patients who achieved histologic response, but not endoscopic response presented with NO narrowing in all segments except for right colon. **.**

Note: Observed data are used for histologic response and endoscopic response.

Endoscopic response is defined as decrease of segmental SES-CD ≥50% from baseline.

Kappa coefficient can be interpreted as follows: ≤0: no agreement; 0.01–0.20: none to slight; 0.21–0.40: fair; 0.41– 0.60: moderate; 0.61–0.80: substantial; 0.81–1.00: almost perfect agreement. (Cohen 1960; McHugh 2012).

CI=confidence interval

# Supplement Table 12. Cut-offs of fecal calprotectin for histologic and combined endoscopic and histologic outcomes^a^

| **Cut-off Values** | **Histologic Response** | **Histologic Remission** | **Histologic-Endoscopic Response** | **Histologic-Endoscopic Remission** |
| --- | --- | --- | --- | --- |
| **Optimal cut-offs**  **(on the original scale, µg/g)** | ≤ 346 | ≤ 158 | ≤ 269 | ≤ 258 |
| **Sensitivity** | 0.721 | 0.691 | 0.758 | 0.951 |
| **Specificity** | 0.622 | 0.693 | 0.622 | 0.571 |
| **Positive predictive value** | 0.743 | 0.506 | 0.585 | 0.331 |
| **Negative predictive value** | 0.595 | 0.831 | 0.785 | 0.981 |
| **Area under the curve** | 0.714 | 0.738 | 0.735 | 0.815 |

^a^Data in the primary analysis set defined as all randomized patients who received ≥1 dose of allocated treatment with baseline SES-CD ≥7 (or ≥4 for isolated ileal disease).

# Supplement Table 13. Descriptive summary of agreement of endoscopic and histologic disease activities at baseline.

| **SES-CD per Segment** | **aRHI=0** | **aRHI>0** | **Total** | **Kappa Coefficient** | **Pearson Correlation** |
| --- | --- | --- | --- | --- | --- |
| **Colon, left** |  |  |  |  | 0.64 (N=1027) |
| SES-CD=0 | 315 (30.67) | 29 (2.82) | 344 (33.50) | 0.46 |  |
| SES-CD>0 | 257 (25.02) | 426 (41.48) | 683 (66.50) |  |  |
| Total | 572 (55.70) | 455 (44.30) | 1027 |  |  |
| **Colon, Rectum** |  |  |  |  | 0.63 (N=1024) |
| SES-CD=0 | 279 (27.25) | 27 (2.64) | 306 (29.88) | 0.38 |  |
| SES-CD>0 | 307 (29.98) | 411 (40.14) | 718 (70.12) |  |  |
| Total | 586 (57.23) | 438 (42.77) | 1024 |  |  |
| **Colon, right** |  |  |  |  | 0.40 (N=936) |
| SES-CD=0 | 163 (17.41) | 36 (3.85) | 199 (21.26) | 0.25 |  |
| SES-CD>0 | 324 (34.62) | 413 (44.12) | 737 (78.74) |  |  |
| Total | 487 (52.03) | 449 (47.97) | 936 |  |  |
| **Colon, transverse** |  |  |  |  | 0.63 (N=990) |
| SES-CD=0 | 383 (38.69) | 50 (5.05) | 433 (43.74) | 0.51 |  |
| SES-CD>0 | 196 (19.80) | 361 (36.46) | 557 (56.26) |  |  |
| Total | 579 (58.48) | 411 (41.52) | 990 |  |  |
| **Ileum** |  |  |  |  | 0.53 (N=865) |
| SES-CD=0 | 147 (16.99) | 49 (5.66) | 196 (22.66) | 0.40 |  |
| SES-CD>0 | 175 (20.23) | 494 (57.11) | 669 (77.34) |  |  |
| Total | 322 (37.23) | 543 (62.77) | 865 |  |  |
| All 5 segments |  |  |  | Pearson Correlation  Baseline  total aRHI | 0.65 (N=1044) |

Numbers are n (%) for SES-CD and aRHI

Left and transverse colons show moderate agreement (K= 0.4632, 0.5134) in endoscopic and histologic disease activities. Other three segments (K=0.3835, 0.2483, 0.3980) show fair agreement between endoscopic and histologic disease activities**.**

Cohen suggested the Kappa result be interpreted as follows: values ≤ 0 as indicating no agreement and 0.01–0.20 as none to slight, 0.21–0.40 as fair, 0.41– 0.60 as moderate, 0.61–0.80 as substantial, and 0.81–1.00 as almost perfect agreement. (Cohen 1960; McHugh 2012)

Pearson Correlation of SES-CD versus aRHI by segment at baseline is interpreted as follows: 0.10 to 0.30 weak; >0.30 to 0.50; moderate; >0.50 to 1 strong; Only patients with observed data at baseline were analyzed. (McHugh ML 2012).

# References

1. D'Haens GR, Geboes K, Peeters M, Baert F, Penninckx F, Rutgeerts P. Early lesions of recurrent Crohn's disease caused by infusion of intestinal contents in excluded ileum. *Gastroenterology*. 1998;114(2):262-267. doi:10.1016/s0016-5085(98)70476-7

2. Magro F, Protic M, De Hertogh G, et al. Effects of Mirikizumab on Histologic Resolution of Crohn's Disease in a Randomized Controlled Phase 2 Trial. *Clin Gastroenterol Hepatol*. 2024;22(9):1878-1888.e10. doi:10.1016/j.cgh.2023.11.010

3. Mosli MH, Parker CE, Nelson SA, et al. Histologic scoring indices for evaluation of disease activity in ulcerative colitis. *Cochrane Database Syst Rev*. 2017;5(5):CD011256. Published 2017 May 25. doi:10.1002/14651858.CD011256.pub2

4. Cohen J. A coefficient of agreement for nominal scales. Educ Psychol Meas 1960;20:37-46.

5. McHugh ML. Interrater reliability: the kappa statistic. *Biochem Med (Zagreb)*. 2012;22(3):276-282.
